# Supplementary figures and images for: Distribution of ESBL-producing and carbapenem-resistant E. coli and Salmonella spp. in retail chicken meat and live bird market sewage in Bangladesh
Source: PLoS One. 2026 Apr 30;21(4):e0347107. doi: 10.1371/journal.pone.0347107 (PMC13132184; doi:10.1371/journal.pone.0347107)

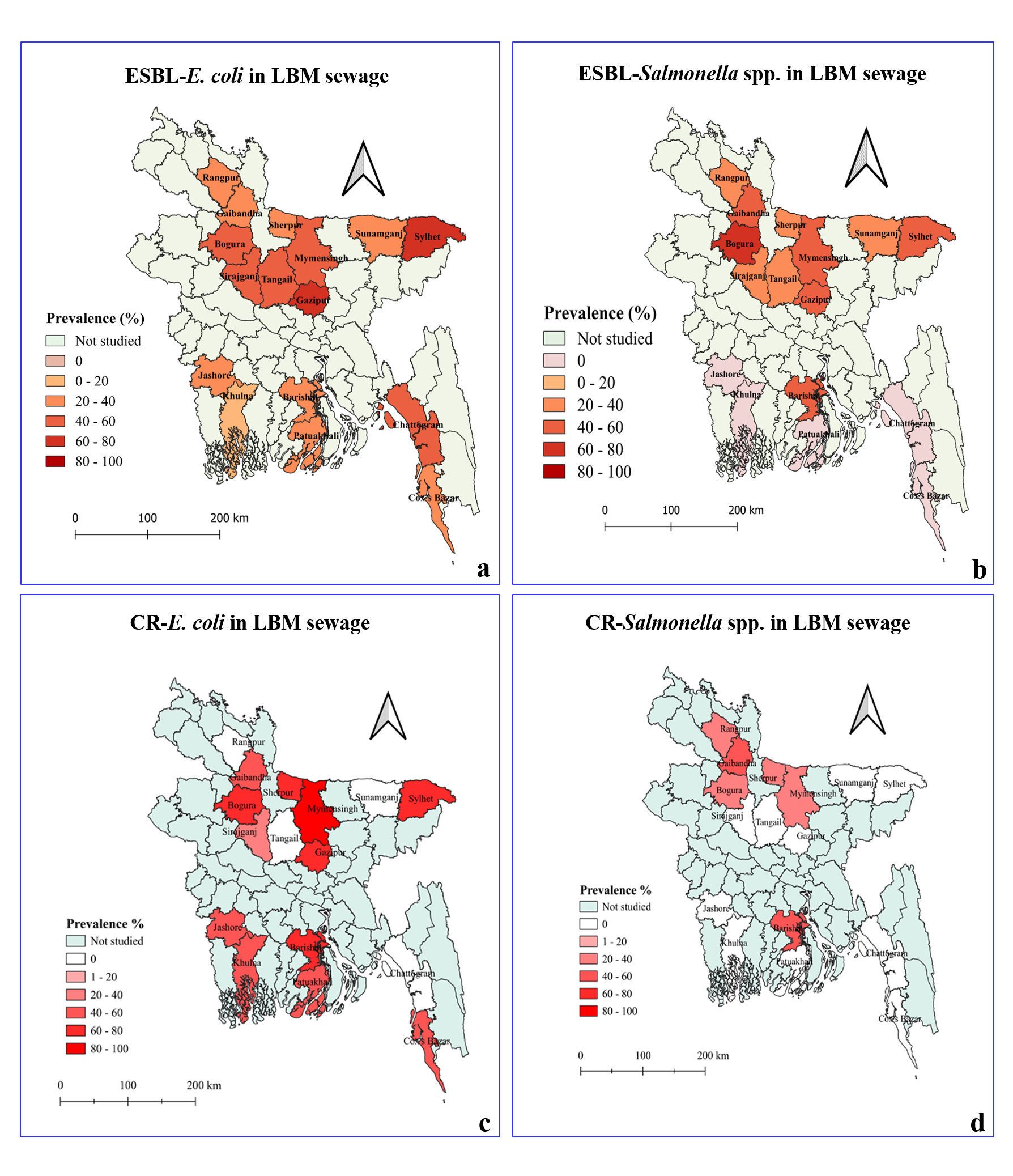

Supplement: S1 Fig — (TIF) [file pone.0347107.s001.tif]

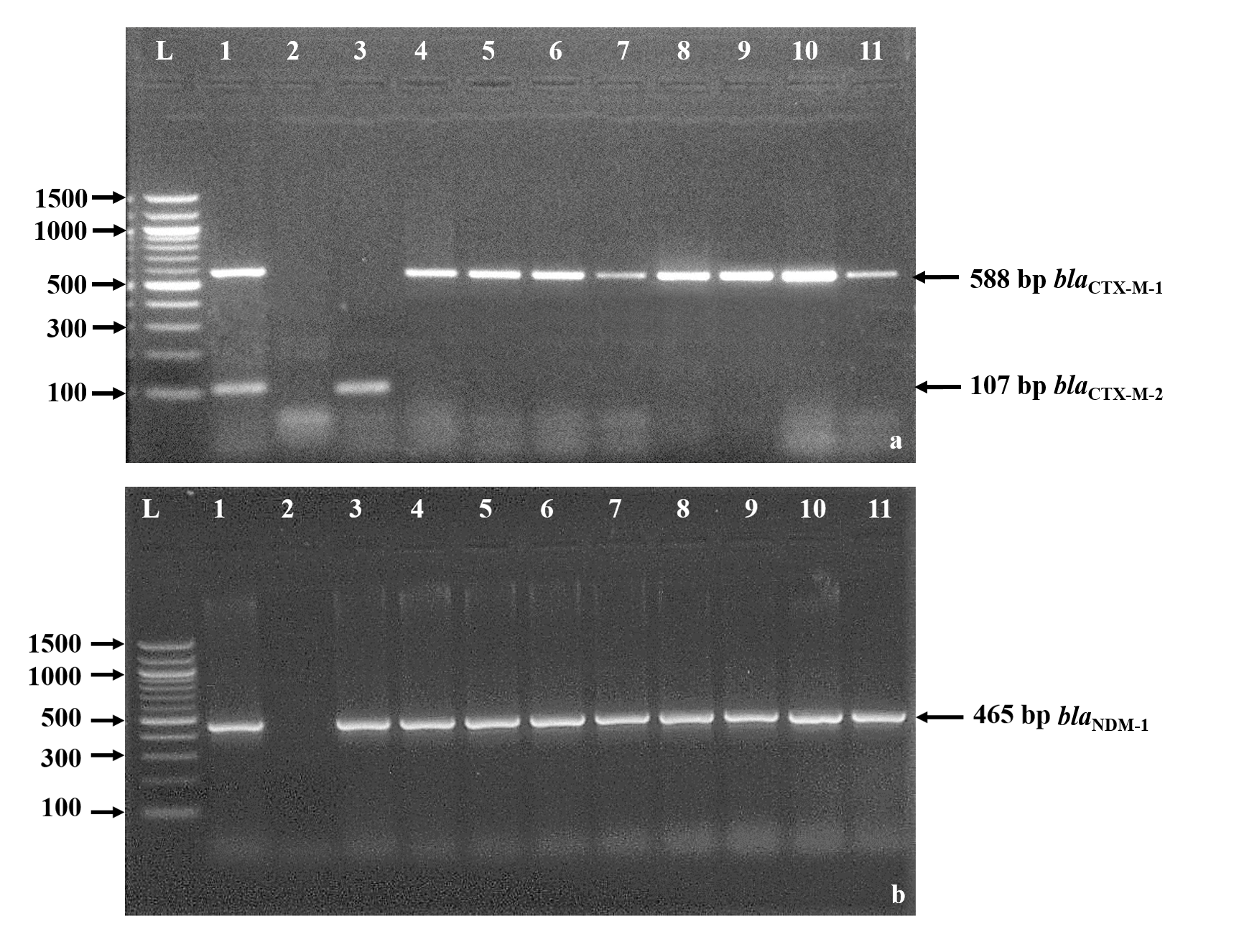

Supplement: S2 Fig — Legends: a) L = DNA marker (100 bp), Lane 1 = Positive control (blaCTX-M-1 and blaCTX-M-2), Lane 2 = Negative control, Lane 3 = Positive for blaCTX-M-2 gene; Lane 4−11 = Positive for blaCTX-M-1 gene. b) L = DNA marker (100 bp), Lane 1 = Positive control (blaNDM-1), Lane 2 = Negative control, Lane 3−11 = Positive for blaNDM-1. (TIF) [file pone.0347107.s002.tif]
