# Supplementary material for: Distribution of ESBL-producing and carbapenem-resistant E. coli and Salmonella spp. in retail chicken meat and live bird market sewage in Bangladesh
Source: PLoS One. 2026 Apr 30;21(4):e0347107. doi: 10.1371/journal.pone.0347107 (PMC13132184; doi:10.1371/journal.pone.0347107)
Supplement: S1 Table — (DOCX) [file pone.0347107.s003.docx]

**S1 Table. District-wise distribution of ESBL-*E. coli*, ESBL-*Salmonella* spp., CR-*E. coli*, and CR-*Salmonella* spp. isolated from retail chicken meat and LBM sewage samples.**

| **Districts** | **Retail chicken meat** | | | | **LBM sewage** | | | |
| --- | --- | --- | --- | --- | --- | --- | --- | --- |
|  | **ESBL-*E. coli* (95% CI)** | **ESBL-*Salmonella* spp. (95% CI)** | **CR-*E. coli* (95% CI)** | **CR-*Salmonella* spp. (95% CI)** | **ESBL-*E. coli* (95% CI)** | **ESBL-*Salmonella* spp. (95% CI)** | **CR-*E. coli* (95% CI)** | **CR-*Salmonella* spp. (95% CI)** |
| Mymensingh | 80 (56-94) | 60 (36-81) | 75 (51-91) | 20 (6-44) | 100 (40-100) | 50 (7-93) | 100 (40-100) | 25 (1-81) |
| Sherpur | 65 (41-85) | 30 (12-54) | 40 (19-64) | 45 (23-68) | 100 (40-100) | 25 (1-81) | 75 (19-99) | 25 (1-81) |
| Bogura | 80 (56-94) | 45 (23-68) | 75 (51-91) | 25 (9-49) | 100 (40-100) | 75 (19-99) | 75 (19-99) | 25 (1-81) |
| Sirajganj | 60 (36-81) | 60 (36-81) | 50 (27-73) | 45 (23-68) | 100 (40-100) | 25 (1-81) | 25 (1-81) | 0 |
| Gazipur | 65 (41-85) | 70 (46-88) | 45 (23-68) | 30 (12-54) | 100 (40-100) | 50 (7-93) | 75 (19-99) | 0 |
| Tangail | 70 (46-88) | 50 (27-73) | 60 (36-81) | 45 (23-68) | 75 (19-99) | 25 (1-81) | 0 | 0 |
| Barishal | 90 (68-99) | 40 (19-64) | 80 (56-94) | 55 (32-77) | 75 (19-99) | 50 (7-93) | 75 (19-99) | 50 (7-93) |
| Patuakhali | 85 (62-97) | 40 (19-64) | 50 (27-73) | 45 (23-68) | 100 (40-100) | 0 | 50 (7-93) | 0 |
| Sylhet | 90 (68-99) | 70 (46-88) | 65 (41-85) | 5 (0.1-25) | 100 (40-100) | 50 (7-93) | 75 (19-99) | 0 |
| Sunamganj | 70 (46-88) | 25 (9-49) | 50 (27-73) | 0 | 75 (19-99) | 25 (1-81) | 0 | 0 |
| Chattogram | 80 (56-94) | 55 (32-77) | 60 (36-81) | 15 (3-38) | 25 (1-81) | 0 | 0 | 0 |
| Cox’s Bazar | 65 (41-85) | 35 (15-59) | 30 (12-54) | 60 (36-81) | 75 (19-99) | 0 | 50 (7-93) | 0 |
| Rangpur | 30 (12-54) | 30 (12-54) | 30 (12-54) | 65 (41-85) | 0 | 25 (1-81) | 0 | 25 (1-81) |
| Gaibandha | 45 (23-68) | 30 (12-54) | 45 (23-68) | 65 (41-85) | 50 (7-93) | 50 (7-93) | 50 (7-93) | 50 (7-93) |
| Khulna | 60 (36-81) | 20 (6-44) | 40 (19-64) | 35 (15-59) | 100 (40-100) | 0 | 50 (7-93) | 0 |
| Jashore | 85 (62-97) | 35 (15-59) | 70 (46-88) | 40 (19-64) | 100 (40-100) | 0 | 50 (7-93) | 0 |
