# Supplementary material for: Distribution of ESBL-producing and carbapenem-resistant E. coli and Salmonella spp. in retail chicken meat and live bird market sewage in Bangladesh
Source: PLoS One. 2026 Apr 30;21(4):e0347107. doi: 10.1371/journal.pone.0347107 (PMC13132184; doi:10.1371/journal.pone.0347107)
Supplement: S2 Table — (DOCX) [file pone.0347107.s004.docx]

**S2 Table. Individual antimicrobial resistance pattern of ESBL-*E. coli*, ESBL-*Salmonella* spp., CR-*E. coli* and CR-*Salmonella* spp. isolated from retail chicken meat and LBM sewage samples.**

| **Antimicrobials** | **ESBL-*E. coli*** | **ESBL-*Salmonella* spp.** | **CR-*E. coli*** | **CR-*Salmonella* spp.** | **ESBL-*E. coli*** | **ESBL-*Salmonella* spp.** | **CR-*E. coli*** | **CR-*Salmonella* spp.** |
| --- | --- | --- | --- | --- | --- | --- | --- | --- |
| Ciprofloxacin | 67.4 | 66.9 | 72.3 | 73.9 | 47.1 | 38.9 | 63.3 | 50 |
| Nalidixic acid | 79.9 | 80.6 | 84.4 | 89.1 | 58.8 | 66.7 | 76.7 | 75 |
| Levofloxacin | 61.6 | 10.1 | 67.1 | 13.4 | 43.1 | 5.6 | 63.3 | 12.5 |
| Norfloxacin | 56.3 | 37.4 | 60.7 | 39.5 | 45.1 | 16.7 | 63.3 | 12.5 |
| Gatifloxacin | 66.1 | 25.2 | 72.3 | 35.3 | 47.1 | 16.7 | 66.7 | 37.5 |
| Pefloxacin | 98.7 | 89.9 | 100 | 99.2 | 94.1 | 83.3 | 100 | 87.5 |
| Ofloxacin | 67 | 35.3 | 72.8 | 47.1 | 54.9 | 22.2 | 73.3 | 37.5 |
| Cephalexin | 20.1 | 25.9 | 20.8 | 12.6 | 13.7 | 0 | 16.7 | 0 |
| Cephradine | 20.1 | 25.2 | 19.1 | 13.4 | 17.6 | 11.1 | 26.7 | 0 |
| Cefuroxime | 7.6 | 18 | 6.9 | 8.4 | 13.7 | 11.1 | 20 | 0 |
| Cefaclor | 13.8 | 23 | 12.7 | 10.1 | 15.7 | 0 | 16.7 | 0 |
| Ceftazidime | 8.5 | 19.4 | 8.7 | 7.6 | 7.8 | 5.6 | 13.3 | 0 |
| Ceftriaxone | 10.3 | 15.8 | 10.4 | 7.6 | 15.7 | 0 | 20 | 0 |
| Cefotaxime | 20.5 | 32.4 | 20.2 | 19.3 | 41.2 | 16.7 | 46.7 | 0 |
| Cefixime | 11.6 | 20.9 | 11 | 9.2 | 11.8 | 11.1 | 20 | 12.5 |
| Cefepime | 7.6 | 15.8 | 7.5 | 5.9 | 13.7 | 0 | 16.7 | 0 |
| Cefoxitin | 2.7 | 9.4 | 3.5 | 7.3 | 2 | 5.6 | 3.3 | 12.5 |
| Ampicillin | 95.5 | 84.9 | 96 | 80.7 | 82.4 | 61.1 | 86.7 | 62.5 |
| Amoxicillin-clavulanic acid | 42.9 | 39.6 | 44.5 | 28.6 | 62.7 | 38.9 | 70 | 12.5 |
| Pipercillin-tazobactam | 5.4 | 4.3 | 5.8 | 3.4 | 3.9 | 0 | 3.3 | 0 |
| Imipenem | 73.2 | 57.6 | - | - | 58.8 | 38.9 | - | - |
| Meropenem | 34.4 | 19.4 | - | - | 31.4 | 16.7 | - | - |
| Colistin | 39.3 | 98.6 | 39.3 | 99.2 | 37.3 | 94.4 | 46.7 | 100 |
| Polymyxin B | 4.9 | 98.6 | 3.5 | 99.2 | 7.8 | 94.4 | 13.3 | 100 |
| Aztreonam | 8.9 | 11.5 | 9.2 | 5.9 | 15.7 | 11.1 | 20 | 12.5 |
| Gentamicin | 18.3 | 31.7 | 17.3 | 30.3 | 13.7 | 16.7 | 13.3 | 25 |
| Amikacin | 16.5 | 15.8 | 16.8 | 6.7 | 9.8 | 0 | 10 | 0 |
| Doxycycline | 75.4 | 98.6 | 75.7 | 95.8 | 60.8 | 83.3 | 73.3 | 87.5 |
| Trimethoprim-sulfamethoxazole | 92 | 89.2 | 93.1 | 88.2 | 86.3 | 72.2 | 90 | 62.5 |
| Tigecycline | 0.4 | 1.4 | 0.6 | 46.2 | 0 | 0 | 0 | 0 |
| Chloramphenicol | 26.8 | 43.2 | 29.5 | 41.9 | 11.8 | 22.2 | 10 | 37.5 |

(-) antimicrobials only used for ESBL-*E. coli* and *Salmonella* spp.
