# Supplementary material for: Distribution of ESBL-producing and carbapenem-resistant E. coli and Salmonella spp. in retail chicken meat and live bird market sewage in Bangladesh
Source: PLoS One. 2026 Apr 30;21(4):e0347107. doi: 10.1371/journal.pone.0347107 (PMC13132184; doi:10.1371/journal.pone.0347107)
Supplement: S1 Raw images — (PDF) [file pone.0347107.s005.pdf]

S1 Raw images. Raw agarose gel electrophoresis images of PCR assays showing ESBL-encoding and carbapenemase genes in ESBL-producing and carbapenem-resistant *E. coli* and *Salmonella* spp.

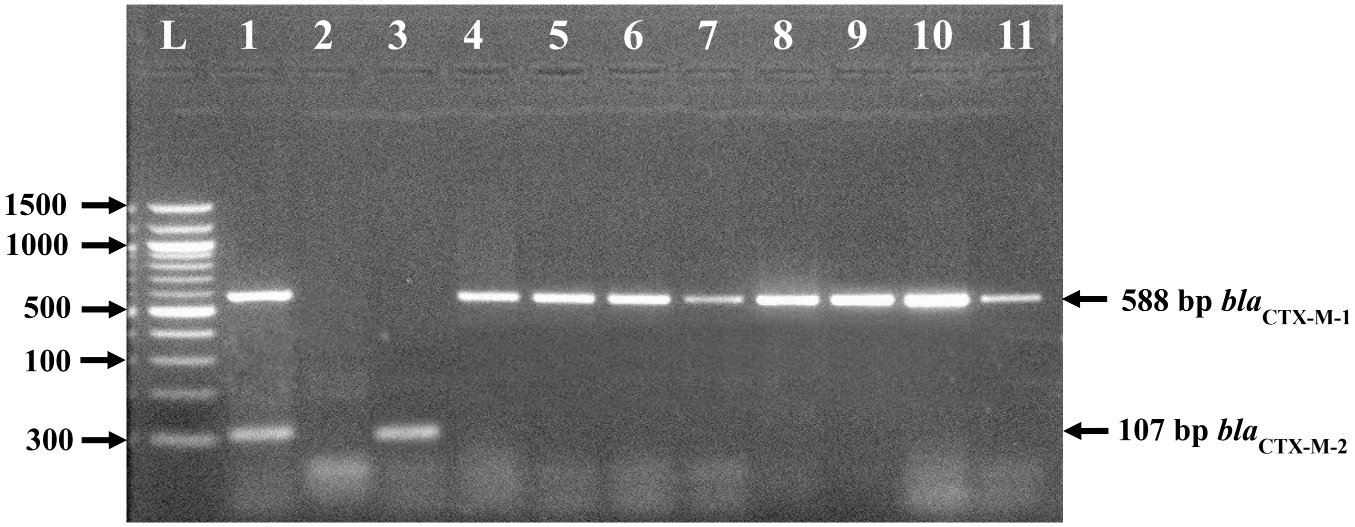

S2a Fig. ESBL-encoding genes in ESBL-producing *E. coli* and *Salmonella* spp. isolates by duplex PCR. PCR amplicons were visualized by using a UV transilluminator and photographed after electrophoresis on 1.5% agarose gel containing 0.5 µg/mL of ethidium bromide. Legends: L = DNA marker (100 bp), Lane 1 = Positive control (*bla*$_{CTX-M-1}$ and *bla*$_{CTX-M-2}$), Lane 2 = Negative control, Lane 3 = Positive for *bla*$_{CTX-M-2}$ gene; Lane 4-11 = Positive for *bla*$_{CTX-M-1}$ gene.

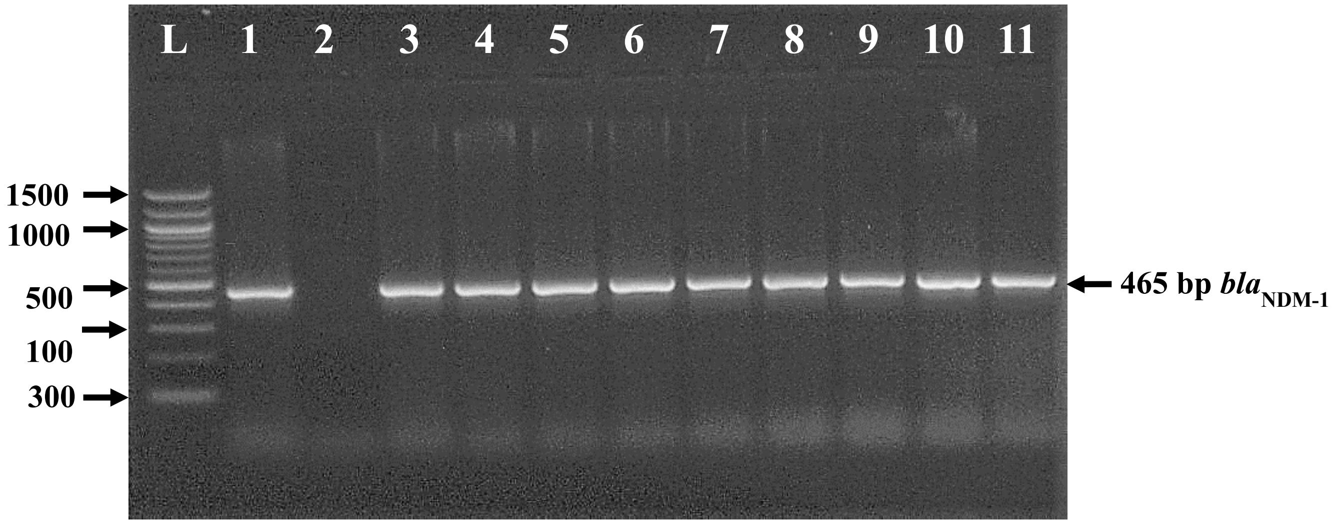

S2b Fig. Carbapenemase genes in CR *E. coli* and *Salmonella* spp. isolates by uniplex PCR. PCR amplicons were visualized by using a UV transilluminator and photographed after electrophoresis on 1.5% agarose gel containing 0.5 µg/mL of ethidium bromide. Legends: L = DNA marker (100 bp), Lane 1 = Positive control ($bla_{\text{CTX-M-1}}$ and $bla_{\text{CTX-M-2}}$), Lane 2 = Negative control, Lane 3 = Positive for $bla_{\text{CTX-M-2}}$ gene; Lane 4-11 = Positive for $bla_{\text{CTX-M-1}}$ gene. b) M = DNA marker (100 bp), Lane 1 = Positive control ($bla_{\text{NDM-1}}$), Lane 2 = Negative control, Lane 3-11 = Positive for $bla_{\text{NDM-1}}$.
