# Supplementary material for: Distribution of ESBL-producing and carbapenem-resistant E. coli and Salmonella spp. in retail chicken meat and live bird market sewage in Bangladesh
Source: PLoS One. 2026 Apr 30;21(4):e0347107. doi: 10.1371/journal.pone.0347107 (PMC13132184; doi:10.1371/journal.pone.0347107)
Supplement: Attachment — Submitted filename: Parvin et al_Response to Reviewers.pdf [file pone.0347107.s008.pdf]

Response to reviewers:

Journal Requirements:
When submitting your revision, we need you to address these additional requirements.
1. Please ensure that your manuscript meets PLOS ONE's style requirements, including those for file naming. The PLOS ONE style templates can be found at https://journals.plos.org/plosone/s/file?id=wjVg/PLOSOne_formatting_sample_main_body.pdf and https://journals.plos.org/plosone/s/file?id=ba62/PLOSOne_formatting_sample_title_authors_affiliations.pdf

Response: We have carefully revised the manuscript to ensure full compliance with PLOS ONE style and formatting requirements, including file naming conventions.

2. In your Methods section, please provide additional information regarding the permits you obtained for the work. Please ensure you have included the full name of the authority that approved the field site access and, if no permits were required, a brief statement explaining why.

Response: Though no specific permission was required for this fieldwork because the study was conducted in live bird markets that are open to the public, and the activities did not involve restricted areas, protected species, or regulated sampling. Verbal consent was taken from mayor of the city corporation or municipality of LBMs (Line 112-119).

3. We noticed you have some minor occurrence of overlapping text with the following previous publication(s), which needs to be addressed:
https://pdfs.semanticscholar.org/9e43/7517937937eb2d3e5b30178ad9c741781997.pdf
https://www.liebertpub.com/doi/10.1089/fpd.2021.0059

Response: We have carefully reviewed the manuscript and revised the relevant sentences by rephrasing them to ensure originality of expression. We confirm that the data, analyses, and conclusions presented in this manuscript are entirely original. The manuscript has now been revised accordingly to minimize textual similarity.

In your revision ensure you cite all your sources (including your own works), and quote or rephrase any duplicated text outside the methods section. Further consideration is dependent on these concerns being addressed.

Response: We have carefully reviewed the entire manuscript to ensure that all sources, including our own previously published works, are appropriately cited wherever relevant. In addition, any instances of duplicated or closely similar text outside the methods section

particularly in the introduction and discussion, have been fully rephrased. No verbatim text remains outside the methods section.

4. Thank you for stating in your Funding Statement:
"This work was partially supported by the BAS-USDA Program in Agriculture and Life Sciences [grant number BAS-USDA PALS LS-18] and Ministry of Science and Technology of Bangladesh [grant number SRG-221102]."

Please provide an amended statement that declares *all* the funding or sources of support (whether external or internal to your organization) received during this study, as detailed online in our guide for authors at http://journals.plos.org/plosone/s/submit-now.

Please also include the statement "There was no additional external funding received for this study." in your updated Funding Statement.

Response: The funding section has been updated (Line 565-569).

Please include your amended Funding Statement within your cover letter. We will change the online submission form on your behalf.

Response: We have added amended funding statement in the cover letter.

5. Thank you for stating the following financial disclosure:
"This work was partially supported by the BAS-USDA Program in Agriculture and Life Sciences [grant number BAS-USDA PALS LS-18] and Ministry of Science and Technology of Bangladesh [grant number SRG-221102]. "
Please state what role the funders took in the study. If the funders had no role, please state: "The funders had no role in study design, data collection and analysis, decision to publish, or preparation of the manuscript."

Response: This statement was already stated in the funding statement section of the original manuscript (Line 567).

If this statement is not correct you must amend it as needed.
Please include this amended Role of Funder statement in your cover letter; we will change the online submission form on your behalf.

6. When completing the data availability statement of the submission form, you indicated that you will make your data available on acceptance. We strongly recommend all authors decide on a data sharing plan before acceptance, as the process can be lengthy and hold up publication timelines. Please note that, though access restrictions are acceptable now, your entire data will need to be made freely accessible if your manuscript is accepted for publication. This policy

applies to all data except where public deposition would breach compliance with the protocol approved by your research ethics board. If you are unable to adhere to our open data policy, please kindly revise your statement to explain your reasoning and we will seek the editor's input on an exemption. Please be assured that, once you have provided your new statement, the assessment of your exemption will not hold up the peer review process.

Response: All relevant data is included in the manuscript and the Supporting Information file.

7. We note that Figure 1, 5 and 6 in your submission contain [map/satellite] images which may be copyrighted. All PLOS content is published under the Creative Commons Attribution License (CC BY 4.0), which means that the manuscript, images, and Supporting Information files will be freely available online, and any third party is permitted to access, download, copy, distribute, and use these materials in any way, even commercially, with proper attribution. For these reasons, we cannot publish previously copyrighted maps or satellite images created using proprietary data, such as Google software (Google Maps, Street View, and Earth). For more information, see our copyright guidelines: http://journals.plos.org/plosone/s/licenses-and-copyright.

Response: All maps now shown in Figures 1, 2a-d and S1 Fig are original, author-generated figures created using open-source QGIS software. No copyrighted, proprietary, or restricted sources (including Google Maps, Google Earth, or similar platforms) were used in their preparation. The shape files used for map generation were obtained from the website of the Humanitarian Data Exchange: (https://data.humdata.org/dataset/cod-ab-bgd).

We require you to either (1) present written permission from the copyright holder to publish these figures specifically under the CC BY 4.0 license, or (2) remove the figures from your submission:

1. You may seek permission from the original copyright holder of Figure 1, 5 and 6 to publish the content specifically under the CC BY 4.0 license.

We recommend that you contact the original copyright holder with the Content Permission Form (http://journals.plos.org/plosone/s/file?id=7c09/content-permission-form.pdf) and the following text:

"I request permission for the open-access journal PLOS ONE to publish XXX under the Creative Commons Attribution License (CCAL) CC BY 4.0 (http://creativecommons.org/licenses/by/4.0/). Please be aware that this license allows unrestricted use and distribution, even commercially, by third parties. Please reply and provide explicit written permission to publish XXX under a CC BY license and complete the attached form."

Response: We did not use any copyright figures.

Please upload the completed Content Permission Form or other proof of granted permissions as an ""Other"" file with your submission.
In the figure caption of the copyrighted figure, please include the following text: “Reprinted from [ref] under a CC BY license, with permission from [name of publisher], original copyright [original copyright year].”

2. If you are unable to obtain permission from the original copyright holder to publish these figures under the CC BY 4.0 license or if the copyright holder’s requirements are incompatible with the CC BY 4.0 license, please either i) remove the figure or ii) supply a replacement figure that complies with the CC BY 4.0 license. Please check copyright information on all replacement figures and update the figure caption with source information. If applicable, please specify in the figure caption text when a figure is similar but not identical to the original image and is therefore for illustrative purposes only.
The following resources for replacing copyrighted map figures may be helpful:
USGS National Map Viewer (public domain): http://viewer.nationalmap.gov/viewer/
The Gateway to Astronaut Photography of Earth (public domain): http://eol.jsc.nasa.gov/sseop/clickmap/
Maps at the CIA (public domain): https://www.cia.gov/library/publications/the-world-factbook/index.html and https://www.cia.gov/library/publications/cia-maps-publications/index.html
NASA Earth Observatory (public domain): http://earthobservatory.nasa.gov/
Landsat: http://landsat.visibleearth.nasa.gov/
USGS EROS (Earth Resources Observatory and Science (EROS) Center) (public domain): http://eros.usgs.gov/#
Natural Earth (public domain): http://www.naturalearthdata.com/

8. PLOS ONE now requires that authors provide the original uncropped and unadjusted images underlying all blot or gel results reported in a submission’s figures or Supporting Information files. This policy and the journal’s other requirements for blot/gel reporting and figure preparation are described in detail at https://journals.plos.org/plosone/s/figures#loc-blot-and-gel-reporting-requirements and https://journals.plos.org/plosone/s/figures#loc-preparing-figures-from-image-files. When you submit your revised manuscript, please ensure that your figures adhere fully to these guidelines and provide the original underlying images for all blot or gel data reported in your submission. See the following link for instructions on providing the original image data: https://journals.plos.org/plosone/s/figures#loc-original-images-for-blots-and-gels.

Response: We confirm that all figures containing blot or gel data in the manuscript have been reviewed to ensure full compliance with PLOS ONE’s blot and gel reporting and figure preparation guidelines. The original, uncropped, and unadjusted image files underlying all blot and gel results have now been provided as S1_raw_images, in accordance with the journal’s

requirements. Adjustments applied to the images presented in the S1a-b Fig were limited to uniform brightness and contrast adjustments.

In your cover letter, please note whether your blot/gel image data are in Supporting Information or posted at a public data repository, provide the repository URL if relevant, and provide specific details as to which raw blot/gel images, if any, are not available. Email us at plosone@plos.org if you have any questions.

Response: Added in cover letter.

Reviewer #1: General Comments
This manuscript addresses an important and timely topic, namely the prevalence of ESBL-producing and carbapenem-resistant E. coli and Salmonella spp. in poultry meat and live bird market (LBM) sewage in Bangladesh. The study is ambitious, covering all eight divisions of the country, and provides valuable epidemiological data with direct relevance for food safety and antimicrobial resistance (AMR) surveillance. The paper is methodologically sound, but the manuscript is lengthy, data-heavy, and at times repetitive. The clarity of presentation can be improved by streamlining results, tightening the introduction and discussion, and emphasizing the key public health implications.

Response: Revised.

Specific comments are provided below.

Abstract
Well done, it is recommended that only the main prevalence figures and key results be highlighted.

Response**:** We have revised the relevant section to present the findings in a more focused and streamlined manner.

Introduction
Well contextualized, but partially repetitive (antibiotic misuse, poultry as reservoir). Condense to improve flow.
Explicitly state the knowledge gap: previous studies in Bangladesh were fragmented, this is the first comprehensive survey covering all divisions.

Response**:** The Introduction has been carefully revised to reduce redundancy.

Methods
Sampling design is solid but justification for "5 samples per LBM" should be added. Clarify whether clustering effects (design effect) were considered in sample size calculation.

Provide more detail on the positive and negative controls used in PCR assays.

Response**:** A justification for collecting five samples per LBM has now been added to the Methods section. In addition, we have clarified the sample size calculation to address clustering effects (Line 131-136).
Detail on the positive and negative controls used in PCR assays have been provided (181-185; 210-212).

Results
Results are highly detailed, with many percentages. Consider moving some data to supplementary materials.
Figures and maps are informative but crowded; merging or simplifying some would enhance readability.
Report confidence intervals along with prevalence estimates.

Response: To improve clarity and readability, we have streamlined the Results section by reducing repetitive percentage reporting and consolidating parallel findings. Detailed resistance profiles and extended percentage data have now been moved to the supporting information section (S1 Fig, S1 Table and S2 Table). In addition, several maps have been simplified by merging overlapping panels and reducing visual complexity, while key findings are retained in the main figures.

Discussion
The discussion is well written, but could perhaps be improved by better comparing more recent studies, if available, from South/South-East Asia and Africa.
Perhaps a clearer distinction could be made between explanations based on concrete evidence (e.g. the use of antibiotics) and more speculative interpretations.

Discuss in more detail the feasibility of the proposed interventions (antibiotic management, LBM sanitation services) in the context of Bangladesh.

Response: We have revised the discussion section.

Conclusion
The conclusions appear appropriate. Nevertheless, it would be advisable to include, among the practical recommendations, the strengthening of biosecurity measures throughout all stages of production and the implementation of stricter drug control. Such measures are consistent with practices adopted in other production systems, where antimicrobials are administered exclusively following a veterinary diagnosis and several active substances are restricted from use in livestock production.

Response: We have revised the conclusion section to explicitly incorporate practical recommendations.

Grammatical or stylistic corrections

Line 55- in the medical, veterinary and agriculture sectors. correct in... medical, veterinary, and agricultural sectors.
Response: Corrected.

Line 57-58 - ...through faces or manure... feces" (non "faces").
Response: Corrected.

Line 59-61 - ...and it is anticipated to climb by 67% by 2030 in rapidly polluting and developing countries around the world.... shorten the thought type.....and is projected to rise by 67% by 2030, particularly in rapidly developing countries."
Response: Corrected.

Line 64–67-"However, the chicken industry's fast expansion, combined with widespread and frequently uncontrolled antibiotic usage, has contributed to the spread of MDR bacteria across the food chain." Better. "The rapid expansion of the chicken industry, combined with uncontrolled antibiotic usage, has facilitated the spread of MDR bacteria through the food chain".
Response: Corrected.

Line 86-87 ...E. coli and Salmonella spp., that produces ESBLs, and demonstrates MDR, has implications..."
correct in... "that produce ESBLs and demonstrate MDR have implications..."
Response: Corrected.

Line 93–94 - "...have demonstrated alarming levels of resistance..." better..high levels (eliminate alarming).
Response: Corrected.

Line 109–110 - "...possible hotspot for ESBL-producing and carbapenem resistant bacteria to humans." Better "hotspot for transmission ... to humans."
Response: Corrected.

Line 523 ....were MDR.." there is a double point
Response: Corrected.

Line 526–535 -"The occurrence of high MDR and existance of pXDR among ESBL-producing and CR-E. coli and Salmonella spp. could be due to widespreed and unrestricted use of antibiotics during poultry production in Bangladesh as well as low levels of waste management

system, which increases selective pressure for MDR bacteria, thus facilitating the emergence and dissemination of MDR foodborne bacteria in poultry production system and retail poultry products.” The sentence is too long. It should be split into at least two clauses.

Line 531–532 .....high MDR and existance of pXDR.... correct as “existence”.
Response: Corrected and splitted.

Line 533....widespread and unrestricted use... correct as “widespread”.
Response: Corrected.

Line 555...ESBL-producing...” correct as. “producing”.
Response: Corrected.

Line 606–608
“The drug regularity authority of Bangladesh should develop an implementable monitoring and evaluation system to tackle this issue. Besides, alternative therapies should be explored and assessed for treating and preventing multidrug resistant bacterial infections.” The sentence can be made more fluid: “The Bangladesh drug regulatory authority should implement effective monitoring systems, and alternative therapies should be explored to treat and prevent MDR infections.”.
drug regularity authority of Bangladesh...correct as “drug regulatory authority...”
Response: Corrected.

Reviewer #2: Comments to authors:
The current study is interesting and clearly describes the findings with strong methedology; however, the authors are recommended to address the following comments to improve the overall quality of the manuscript:

Abstract
Clear and well-written

Introduction
1. Check for punctuation errors throughout the manuscript (double space, eg, line 60, comma eg. line 65)
Response: Corrected.

2. Line 69. Among MDR strains, foodborne pathogens, especially Escherichia coli (E. coli) ____. The abbreviation should be introduced in the sentences above before using the short form
Response: Corrected.

3. Line 82. Please cross-check that all scientific gene names are written correctly throughout the manuscript, eg. if CTX-M has to be in subscript format (blaCTX-M) Method and material

Response: All gene names have been replaced throughout the manuscript.

4. Line 136-137. A cross-sectional live bird market (LBM) survey was carried out on 64 LBMs of 32 upazilas in 16 districts (second tier of administrative regions) of Bangladesh. For better clarity, it is recommended to rewrite the sentence. Here are the suggested revisions: A cross-sectional survey of live bird markets (LBM) was conducted in 64 LBM locations across 32 upazilas in 16 districts (the second tier of administrative regions) of Bangladesh
Response: Corrected.

5. Line 154-155. Prior to collection of samples, the informed consent was taken from the chicken's seller.
I recommend rewriting this sentence, here is the suggested: Prior to the collection of samples, informed consent was taken from the chicken seller.
Response: Corrected.

6. Line 212-213. The presence of ESBLs-encoding genes (blaCTX-M-1, and blaCTX-M-2) in ESBL-producing E. coli and Salmonella spp. isolates was determined by duplex PCR using specific oligonucleotide primers[35]

Question. 1. Why did you choose to screen only these ESBL genes? Why not include blaTEM and blaSHV as well?

Response: Thank you for this thoughtful question. The decision to focus on this specific set of ESBL genes was based on a combination of epidemiological relevance, local resistance patterns, and the scientific objectives of the study. On the other hand, ESBL genes mainly blaCTX-M-1, blaCTX-2 represents the most frequently encountered ESBL determinants in clinical isolates worldwide, particularly within Enterobacterales. Although blaTEM and blaSHV were historically the predominant ESBL genes, recent surveillance data and local antimicrobial resistance reports consistently show that CTX-M–type ESBLs now account for the overwhelming majority of ESBL-mediated resistance in our setting.

Question. 2. Why did you choose to screen only one Cr gene?
Response: Our decision to screen only one carbapenem-resistance (blaNDM-1) gene was designed to focus on the most epidemiologically relevant carbapenem-resistance determinant in our setting, which based on local surveillance reports, published regional data, and routine clinical diagnostics has consistently been the predominant mechanism of carbapenem resistance among the isolates we encounter.

7. line 230-231………… commercially available imipenem (10 µg) and meropenem (10 µg) disks. Isolates that were resistant to at least one carbapenem drug were considered as CR isolates[34, 36], …..

These sentences contain wording errors and should be corrected: disk must be disks, …….on=one
Response: Corrected.

8. Line 236-239. The PCR reaction mixtures of 25 µL total volume containing 12.5 µL of OneTaq® Quick-Load® 2X PCR Master Mix with standard buffer (New England, BioLabs Inc.), 8.5 µL water, 1 µL template DNA from each isolate, and 1.5 µL from each primer with a concentration of 15 pmol.
For better clarity, these sentences should be rewritten. You may consider the following suggested revisions.
The PCR reaction mixtures, with a total volume of 25 µL, contained 12.5 µL of OneTaq® Quick-Load® 2X PCR Master Mix with standard buffer (New England Biolabs Inc.), 8.5 µL of water, 1 µL of template DNA from each isolate, and 1.5 µL of each primer at a concentration of 15 pmol.
Response: Corrected.

Result

9. Line 316-317. The carbapenemase gene, blaNDM-1, was detected in 5.2% (9/173) isolates of CR-E. coli and 33.6% (40/119) isolates of CR-Salmonella spp Suggested correction The carbapenemase gene, blaNDM-1, was detected in 5.2% (9/173) of CR-E. coli isolates and 33.6% (40/119) isolates of CR-Salmonella spp.
Response: Corrected.

10. Line 391-393. Isolates of ESBL-E. coli (40.6% and 21%) and Salmonella spp. (34.5% and 22.3%), as well as CR-E. coli (42.8% and 21.4%) and Salmonella spp. (42.9% and 25.2%) recovered from retail chicken meat, exhibited resistance to 9–12 and 5–8 antimicrobial agents, respectively
These sentences are unclear. What do the two percentages in one bracket represent (e.g., 40.6% and 21%; 34.5% and 22.3%)? Please rewrite them to avoid confusion for readers.
Response: Corrected.

Discussion

11. Line 486. Interestingly, the blaCTX-M-2 gene was only detected in a single (2%) E. coli Instead of using a word single … only one, suggested correction. Interestingly, the blaCTX-M-2 gene was detected in only one (2%) E. coli
Response: Corrected.

12. Line 516-518. Since different LBMs are anticipated to use different management techniques while slaughtering of chickens, and processing of chicken meat, there are varying hazards

Suggested corrections. Since different LBMs are anticipated to use different management techniques during the slaughter of chickens and processing of chicken meat, varying hazards are associated with the presence of CR-E. coli and Salmonella spp.

Response: Corrected.

13. Line 570-571 Additionally, Bangladesh should control the use of antibiotics in poultry in order to reduce the levels of resistance found in the current study.

Suggested correction. Additionally, Bangladesh should regulate the use of antibiotics in poultry to reduce the levels of resistance found in the current study.

Response: Corrected.

14. In the discussion section, please include the public health implications of the high prevalence of MDR, ESBL-producing E. coli and Salmonella spp., as well as carbapenem-resistant E. coli and Salmonella spp. detected in sewage samples, and explain their potential role in disseminating antimicrobial resistance (AMR) into the environment

Response: Added.
