# Supplementary material for: Distribution of ESBL-producing and carbapenem-resistant E. coli and Salmonella spp. in retail chicken meat and live bird market sewage in Bangladesh
Source: PLoS One. 2026 Apr 30;21(4):e0347107. doi: 10.1371/journal.pone.0347107 (PMC13132184; doi:10.1371/journal.pone.0347107)
Supplement: Attachment — Submitted filename: plosOnepaper1.docx [file pone.0347107.s006.docx]

Comments to authors:

The current study is interesting and clearly describes the findings with strong methodology; however, the authors are recommended to address the following comments to improve the overall quality of the manuscript:

**Abstract**

Clear and well-written

**Introduction**

1. Check for punctuation errors throughout the manuscript (double space, eg, line 60, comma eg. line 65)
2. Line 69. Among MDR strains, foodborne pathogens, especially *Escherichia coli* (*E. coli*) ____. *The abbreviation should be introduced in the sentences above before using the short form*
3. Line 82. Please cross-check that all scientific gene names are written correctly throughout the manuscript, eg. if CTX-M has to be in **subscript** format (*bla_CTX-M_*)

**Method and material**

4. Line 136-137. A cross-sectional live bird market (LBM) survey was carried out on 64 LBMs of 32 upazilas in 16 districts (second tier of administrative regions) of Bangladesh.

For better clarity, it is recommended to rewrite the sentence. Here are the suggested revisions: *A cross-sectional survey of live bird markets (LBM) was conducted in 64 LBM locations across 32 upazilas in 16 districts (the second tier of administrative regions) of Bangladesh*

5. Line 154-155. Prior to collection of samples, the informed consent was taken from the chicken’s seller.

I recommend rewriting this sentence, here is the suggested: *Prior to the collection of samples, informed consent was taken from the chicken seller.*

6. Line 212-213. The presence of ESBLs-encoding genes (*bla*CTX-M-1, and *bla*CTX-M-2) in ESBL-producing *E. coli* and *Salmonella* spp. isolates was determined by duplex PCR using specific oligonucleotide primers[35]

*Question. 1.* Why did you choose to screen only these ESBL genes? Why not include *bla_TEM_* and *bla_SHV_* as well?

*Question.* 2. Why did you choose to screen only one Cr gene?

7. line 230-231.…………commercially available imipenem (10 µg) and meropenem (10 µg) disks. Isolates that were resistant to at least one carbapenem drug were considered as CR isolates[34, 36], …..

*These sentences contain wording errors and should be corrected: disk must be disks, …….on=one*

8. Line 236-239. The PCR reaction mixtures of 25 µL total volume containing 12.5 µL of OneTaq^®^ Quick-Load^®^ 2X PCR Master Mix with standard buffer (New England, BioLabs Inc.), 8.5 µL water, 1 µL template DNA from each isolate, and 1.5 µL from each primer with a concentration of 15 pmol.

For better clarity, these sentences should be rewritten. You may consider the following suggested revisions.

*The PCR reaction mixtures, with a total volume of 25 µL, contained 12.5 µL of* OneTaq^®^ Quick-Load^®^ *2X PCR Master Mix with standard buffer (New England Biolabs Inc.), 8.5 µL of water, 1 µL of template DNA from each isolate, and 1.5 µL of each primer at a concentration of 15 pmol.*

**Result**

9. *Line 316-317.* The carbapenemase gene, *bla*NDM-1, was detected in 5.2% (9/173) isolates of CR-*E. coli* and 33.6% (40/119) isolates of CR-*Salmonella* spp

*Suggested correction* The carbapenemase gene, *bla*NDM-1, was detected in 5.2% (9/173) of CR-*E. coli* isolates and 33.6% (40/119) isolates of CR-*Salmonella* spp.

10. Line 391-393. Isolates of ESBL-*E. coli* (40.6% and 21%) and *Salmonella* spp. (34.5% and 22.3%), as well as CR-*E. coli* (42.8% and 21.4%) and *Salmonella* spp. (42.9% and 25.2%) recovered from retail chicken meat, exhibited resistance to 9–12 and 5–8 antimicrobial agents, respectively

*These sentences are unclear. What do the two percentages in one bracket represent (e.g., 40.6% and 21%; 34.5% and 22.3%)? Please rewrite them to avoid confusion for readers.*

**Discussion**

11. Line 486. Interestingly, the *bla*CTX-M-2 gene was only detected in a single (2%) *E. coli*

*Instead of using a word single … only one,*

*suggested correction. Interestingly, the blaCTX-M-2 gene was detected in only one (2%) E. coli*

12. *Line 516-518.* Since different LBMs are anticipated to use different management techniques while slaughtering of chickens, and processing of chicken meat, there are varying hazards

*Suggested corrections**. Since different LBMs are anticipated to use different management techniques during the slaughter of chickens and processing of chicken meat, varying hazards are associated with the presence of CR-E. coli and Salmonella spp*

13. Line 570-571 Additionally, Bangladesh should control the use of antibiotics in poultry in order to reduce the levels of resistance found in the current study.

*Suggested correction. Additionally, Bangladesh should regulate the use of antibiotics in poultry to reduce the levels of resistance found in the current study.*

14. In the discussion section, please include the public health implications of the high prevalence of MDR, ESBL-producing *E. coli* and *Salmonella* spp., as well as carbapenem-resistant *E. coli* and *Salmonella* spp. detected in sewage samples, and explain their potential role in disseminating antimicrobial resistance (AMR) into the environment
