# Supplementary material for: Distribution of ESBL-producing and carbapenem-resistant E. coli and Salmonella spp. in retail chicken meat and live bird market sewage in Bangladesh
Source: PLoS One. 2026 Apr 30;21(4):e0347107. doi: 10.1371/journal.pone.0347107 (PMC13132184; doi:10.1371/journal.pone.0347107)
Supplement: Attachment — Submitted filename: Parvin et al_Manuscript.docx [file pone.0347107.s007.docx]

Distribution of ESBL-producing and carbapenem-resistant *E. coli* and *Salmonella* spp. in retail chicken meat and live bird market sewage in Bangladesh

Mst. Sonia Parvin, Sudipta Talukder, Sayra Tasnin Sharmy, Md. Mehedi Hasan and Md. Taohidul Islam^*^

Population Medicine and AMR Laboratory, Department of Medicine, Faculty of Veterinary Science, Bangladesh Agricultural University, Mymensingh-2202, Bangladesh

^*^**Corresponding author:**

E-mail: [taohid@bau.edu.bd](mailto:taohid@bau.edu.bd) (MTI)

**Short title:** Antibiotic-resistant *E. coli* and *Salmonella* spp. in retail chicken meat and LBM sewage

**Abstract**

A cross-sectional live bird market (LBM) survey was conducted to determine the prevalence and distribution of extended-spectrum β-lactamase (ESBL)-producing and carbapenem-resistant (CR) *E. coli* and *Salmonella* spp. in retail chicken meat and LBM sewage in Bangladesh. *E. coli* and *Salmonella* spp. were identified using culture-based and molecular methods. Isolates were tested for CR by a disk diffusion test; a confirmatory ESBL screening was performed by double disk synergy test. The isolates were screened for ESBL and CR genes using PCR. Prevalence of ESBL-*E. coli* and *Salmonella* spp. in retail chicken meat was 70% and 43.4%, respectively while in LBM sewage, it was 79.7% and 28.1%, respectively. Moreover, CR was detected in 54.1% and 46.9% of *E. coli*, and 37.2% and 12.5% of *Salmonella* spp. isolated from retail chicken meat and LBM sewage, respectively. Molecular analysis revealed the presence of ESBL and CR genes, including *bla*CTX-M-1, *bla*CTX-M-2, and *bla*NDM-1. Multidrug resistance (MDR) was observed in 98.2% and 92.2% of ESBL-*E. coli*, and 97.8% and 94.4% of ESBL-*Salmonella* spp. from retail chicken meat and LBM sewage, respectively. However, 98.8% and 100% of CR-*E. coli*, and 97.5% and 87.5% of CR-*Salmonella* spp. from both types of samples, respectively were MDR. These results highlight the urgent need for improved antibiotic stewardship, regular surveillance, and biosecurity measures in poultry production and LBM sanitation to mitigate the risks associated with antimicrobial resistance in Bangladesh.

**Keywords:** ESBL-producing and CR *E. coli* and *Salmonella* spp.; MDR; Resistance genes, Retail chicken meat; LBM sewage; Bangladesh

**Introduction**

Antimicrobial resistance (AMR) has emerged as a critical global concern in the twenty-first century in humans, animals, and the environment[1]. The primary factor contributing to the global growth of AMR may be the improper use and inadequate regulations, lack of awareness leading to excessive or inept antibiotic use in the medical, veterinary and agriculture sectors. The issue has gotten worse due to careless over-the-counter antibiotic sales, poor sanitation practices, and the release of unmetabolized antibiotics or their residues into the environment through faces or manure, and the use of antibiotics as growth promoters in poultry and livestock rather than as agents to control infections[2]. Recently, antibiotic usage in livestock and poultry production has increased at an unprecedented rate across continents, and it is anticipated to climb by 67% by 2030 in rapidly polluting and developing countries around the world[3]. Excessive and inconsistent use of antimicrobials in livestock and poultry production has led to the emergence of multidrug-resistant (MDR) bacterial pathogens in the community that can spread AMR globally[2]. The global dissemination of MDR strains, especially in low- and middle-income countries like Bangladesh, where antimicrobial use in livestock and poultry production is less regulated, exacerbates this issue[2]. However, the chicken industry's fast expansion, combined with widespread and frequently uncontrolled antibiotic usage, has contributed to the spread of MDR bacteria across the food chain[4, 5]. Among MDR strains, foodborne pathogens, especially *Escherichia coli* (*E. coli*) and *Salmonella* spp. are particularly concerning due to their zoonotic potential and ability to acquire and distribute resistance genes throughout ecosystems[6].

Poultry meat is one of the most popular animal protein sources worldwide, providing an economical and accessible choice for many people. However, its role as a reservoir for AMR bacteria —particularly *E. coli* and *Salmonella* spp.—has raised public health concerns. These pathogens, commonly seen in retail chicken meat, have demonstrated increasing levels of resistance to critically important antimicrobials, including extended-spectrum β-lactams and carbapenems[7, 8]. Extended-spectrum β-lactamase (ESBL)-producing and carbapenem-resistant *Enterobacterales* (CRE) have emerged globally, raising concerns about food safety and public health[9, 10].

ESBLs are class A beta-lactamases, and are plasmid-mediated enzymes that can hydrolyze a wide range of β-lactam antibiotics, making many frontline treatments ineffective[10]. ESBL genes, mainly *bla*CTX-M genes, often located on plasmids, facilitate horizontal gene transfer among bacteria in the gut of animals and humans, as well as in the broader environment[10]. MDR among ESBL-expressing strains is complicated and influenced by the location of resistance genes on integrons with promoters that coordinate the expression of downstream resistance cassettes[10, 11]. *E. coli* and *Salmonella* spp., that produces ESBLs, and demonstrates MDR, has implications for both human and poultry health[11, 12]. Multidrug resistant ESBL-producing *E. coli* and *Salmonella* spp. have been increasingly isolated from retail chicken meat, indicating that the food chain is an important route for transmission from animals to humans[8]. In addition to *E. coli* and *Salmonella* spp., other MDR organisms, particularly members of the ESKAPE group such as *Klebsiella pneumoniae*, *Pseudomonas aeruginosa*, and *Acinetobacter baumannii*, have emerged as critical contributors to the global AMR crisis. These pathogens are frequently implicated in nosocomial infections and have demonstrated alarming levels of resistance to carbapenems and other last-resort antibiotics[13]. Therefore, a one-health approach is required to control the emergence and effects of multidrug resistant ESBL-producing *E. coli* and *Salmonella* spp.

Carbapenem-resistant *Enterobacterales* have been detected in poultry, which is of particular concern. Although carbapenems are not commonly used in food-producing animals, resistance has emerged due to co-selection mechanisms and environmental contamination[14, 15]. Carbapenemase-producing *E. coli* and *Salmonella* spp. harbor genes such as *bla*KPC, *bla*NDM-1, and *bla*OXA-48, which confer resistance to this last-resort class of antibiotics, posing potential health risk in humans[16].

Live bird markets (LBMs), also known as wet markets, are widespread around the world, including Bangladesh. Typically, they are at the center of an intricate trading network that brings together a diverse range of bird species produced by many farmers from various parts of the country utilizing a variety of poultry production strategies[17]. Aside from providing a variety of live birds, meat, and eggs for human consumption to customers from various ethnic, religious, and cultural backgrounds, bird slaughtering remains are frequently sold as food for animal production, including poultry production[17]. These activities alone make LBM a possible hotspot for ESBL-producing and carbapenem resistant bacteria to humans. Resistant strains can be easily passed from chickens to humans through consumption of contaminated raw or undercooked chicken meat, close chickens contact, and exposure to environmental runoff[11]. In this context, ESBL-producing and carbapenem resistant *E. coli* and *Salmonella* spp. in retail chicken meat are of concern, with implications for human consumers, the health of animals, and the environment[12, 18, 19]. Furthermore, the LBM in Bangladesh is usually regarded as having inadequate hygiene and biosecurity, making it easy for AMR infections to spread through direct animal contact and contaminated settings, such as sewage[5]. Surveillance studies in various regions have reported widespread contamination of retail chicken meat with MDR *E. coli* and *Salmonella* spp., including strains resistant to cephalosporins and carbapenems[20]. These findings highlight the urgent need for integrated monitoring systems, stricter antimicrobial stewardship in animal production, and enhanced food safety protocols. Limited studies conducted in Bangladesh regarding ESBL-producing *E. coli* and *Salmonella* spp. isolated from frozen chicken meat[15, 21], ESBL-*E. coli* from fecal samples and cloacal swab of poultry[22, 23] and drinking water[24-26]. To our knowledge, no comprehensive works (population study) on ESBL-producing and carbapenem resistant *E. coli* and *Salmonella* spp. and their distribution in retail chicken meat and LBM sewage covering all divisions have been conducted in Bangladesh. Therefore, an extensive study is required to have a complete picture of ESBL-producing and carbapenem resistant *E. coli* and *Salmonella* spp. from retail chicken meat, and LBM sewage covering wide areas in Bangladesh. The present study aimed to determine the prevalence and distribution of ESBL-producing and carbapenem-resistant *E. coli* and *Salmonella* spp. in retail chicken meat and live bird market sewage in eight divisions of Bangladesh.

**Methods**

**Study sites and sampling procedure**

A cross-sectional live bird market (LBM) survey was carried out on 64 LBMs of 32 upazilas in 16 districts (second tier of administrative regions) of Bangladesh. There are eight divisions (first tier of administrative regions) in Bangladesh. A multistage random sampling technique was used to select the LBMs. Firstly, two districts from each of the eight divisions, and then two upazilas from each selected district were randomly selected (Fig 1). Finally, two LBMs were randomly selected from each upazila. The list of the LBMs was collected from the respective municipal authority.

**Fig 1. Map showing selected live bird markets (•) in 32 upazilas (orange highlighted areas) of 16 districts (light yellow highlighted areas) of Bangladesh.** [The map was generated by using QGIS 3.34.8 'Prizren' for desktop (qgis.org)].

The sample size was calculated using the formula, n = Z^2^P (1-P)/d^2^, where n = sample size, P = expected prevalence, d = required precision of 5%, Z = 1.96 at 95% confidence level[27]. The expected prevalence of *E. coli* and *Salmonella* spp. in retail chicken meat used for calculating the sample size was 85% and 70%, respectively[28, 29]. These assumptions produced a sample size of 196 and 323, respectively. Therefore, a total of 320 retail chicken meat samples with five samples from each LBM were sampled. Besides, a total of 64 sewage samples having one from each respective LBM were collected. Prior to collection of samples, the informed consent was taken from the chicken’s seller.

**Collection, transportation and processing of samples**

**Meat samples**

Collected meat samples were maintained cool chain in thermal boxes and transported immediately to the laboratory, and processed on the same day. The preparation of meat samples was based on the European standard ISO-16654:2001[30]. During processing, the meat surface was sterilized by stabbing with a hot spatula and the upper portion of meat was removed carefully. Aseptically, 25 g of each meat sample was chopped into very small fine pieces, homogenized for two minutes with 225 mL of buffered peptone water (BPW), and incubated at 37°C for 18 h. Subsequently, 1 mL of each diluted meat sample was added into two separate test tubes; one containing the nutrient broth (NB) for isolation of *E. coli*, and another one containing Rappaport-Vassiliadis Soya broth (RVS) for isolation of *Salmonella* spp., and incubated further for 18 h at 37°C for selective enrichment.

**Sewage water samples**

Sewage water sample (~15 mL) was taken aseptically in a sterile falcon tube. Bacteria were pelleted by centrifugation at 600 × g for 20 min, and re-suspended in 1 mL of BPW and incubated at 37°C for 18 h for pre-enrichment of bacteria. After that, 1 mL of this suspension was incubated in NB and RVS cultures by using broth enrichment as described for the meat samples.

**Bacteriological culture, isolation, and confirmation**

After selective enrichment, a loopful of NB culture was streaked onto Eosin Methylene Blue (EMB) agar in duplicate for the isolation of *E. coli*, while a loopful of the RVS culture was streaked onto Xylose-Lysine-Deoxycholate (XLD) agar in duplicate for the isolation of *Salmonella* spp., and incubated at 37°C for 18–24 h. Typical colonies having dark blue color with a characteristic metallic sheen on EMB, and a black center and a slightly transparent zone of reddish colour on XLD were presumptive for *E. coli*, and *Salmonella* spp., respectively. Three presumptive *E. coli* and *Salmonella* spp. colonies from each selective agar plate were picked, and then subcultured to obtain a pure culture. Gram staining and biochemical tests such as catalase, oxidase, indole, methyl red, Voges–Proskauer tests, a sugar fermentation test using triple sugar iron agar were performed from the pure culture.

Biochemically positive *E. coli* and *Salmonella* spp. isolates were then subjected to DNA extraction using the boiling method for confirmation of the bacteria by polymerase chain reaction (PCR) assay[31], and it was quantified using nanodrop spectrometer (NanoDrop One, Thermo Fisher Scientific, USA). Two uniplex PCR targeting *malB* promoter gene and *ITS* gene were used for the confirmation of *E. coli* and *Salmonella* spp. genus, respectively. Primers used for *E. coli* were ECO-1 (5'-GACCTCGGTTTAGTTCACAGA-3') and ECO-2 (5'-CACACGCTGACGCTGACCA-3') for the amplification of 585 bp[32]. The oligonucleotide primer sequences used for *Salmonella* spp. were ITSF (5′- TATAGCCCCATCGTGTAGTCAGAAC-3′) and ITSR (5′-TGCGGCTGGATCACCTCCTT-3′) with fragment size 312 bp[33]. Amplification reactions and PCR conditions were used in accordance to earlier study[15, 21]. After PCR confirmation, each *E. coli* and *Salmonella* spp. isolates was stored at –20°C with 50% (v/v) glycerol for further testing.

**Screening of extended-spectrum β-lactamases (ESBL)**

Screening of ESBL production for all PCR positive *E. coli* and *Salmonella* spp. isolates were performed by the double disk synergy test[34] employing commercially available four antibiotic disks, namely amoxicillin-clavulanic acid (30 μg), cefotaxime (30 μg), ceftazidime (30 μg), and ceftriaxone (30 μg), which were placed 30 mm apart with amoxicillin-clavulanic acid disk in the middle. After overnight incubation at 37°C, the phenotypic presence of ESBL-producing *E. coli* and *Salmonella* spp. was assessed when the zone of inhibition of any of the extended-spectrum β-lactam disk expanded by at least 5 mm close to amoxicillin-clavulanic acid disk. *E. coli* ATCC 25922 and *Salmonella* Enteritidis ATCC 13076 were used as quality control bacteria.

The presence of ESBLs-encoding genes (*bla*CTX-M-1, and *bla*CTX-M-2) in ESBL-producing *E. coli* and *Salmonella* spp. isolates was determined by duplex PCR using specific oligonucleotide primers[35]. Primers used for *bla*CTX-M-1: ctxm1-15F (5′-GAATTAGAGCGGCAGTCGGG-3′) and ctxm1-02R (5′- CACAACCCAGGAAGCAGGC-3′) for the amplification of 588 bp; for *bla*CTX-M-2: ctxm2-39F (5′-GATGGCGACGCTACCCC-3′) and ctxm2-45R (5′- CAAGCCGACCTCCCGAAC-3′) for the amplification of 107 bp. The PCR reaction mixtures were prepared from 12.5 µL OneTaq^®^ Quick-Load^®^ 2X PCR Master Mix with standard buffer (New England, BioLabs Inc.), 1 μL of each of the forward and reverse primers with a concentration of 10 pmol, 1 µL template DNA from each isolate, and 7.5 μL of nuclease-free water to reach a total volume of 25 µL. The PCR conditions used were as follows: initial denaturation at 95°C for 5 min, followed by 25 cycles of denaturation at 95°C for 30 s, annealing at 60°C for 1 min, and extension at 72°C for 1 min, with a final extension at 72°C for 10 min; then, the PCR products were electrophoresed on a 1.5% agarose gel.

**Screening of carbapenem-resistant isolates**

Carbapenem resistance in *E. coli* and *Salmonella* spp. was screened by the disk diffusion method on Mueller-Hinton agar plates using commercially available imipenem (10 µg) and meropenem (10 µg) disk. Isolates that were resistant to at least on carbapenem drug were considered as CR isolates[34, 36], and selected for the detection of the carbapenemase genes using PCR.

For the detection of carbapenemase-encoding gene *bla*NDM-1, a uniplex PCR was performed using specific oligonucleotide primers as described earlier[37]. The primer sequences were NDM-1 forward: 5′-CTTCCAACGGTTTGATCGTC-3′ and NDM-1 reverse: 5′-TAGTGCTCAGTGTCGGCATC-3′ with a fragment size of 465 bp. The PCR reaction mixtures of 25 µL total volume containing 12.5 µL of OneTaq^®^ Quick-Load^®^ 2X PCR Master Mix with standard buffer (New England, BioLabs Inc.), 8.5 µL water, 1 µL template DNA from each isolate, and 1.5 µL from each primer with a concentration of 15 pmol. PCR was run with an initial denaturation at 95°C for 7 min; 30 cycles of 95°C for 1 min, 55°C for 1 min, and 68°C for 1 min; and a final elongation at 68°C for 7 min. The PCR product was electrophoresed on a 1.5% agarose gel to determine the size of the product. Negative control was included in all reactions, which contained all the components of the PCR mixture, but with water instead of the template DNA. In addition, a 100 bp DNA ladder (Size range: 100-1000 bp, New England, BioLabs Inc.) was run simultaneously to detect the size of the bands.

**Antimicrobial susceptibility testing**

The antimicrobial susceptibility testing of all ESBL- producing and CR-*E. coli* and *Salmonella* spp. isolates was carried out against 29 different antimicrobials representing 14 distinct antimicrobial classes by Kirby-Bauer disk diffusion method according to the guidelines recommended by the Clinical and Laboratory Standard Institute (CLSI)[34]. For colistin and polymyxin B, minimum inhibitory concentrations (MICs) were determined by broth microdilution method. The interpretation of the results was based on the guidelines of CLSI[34], and in some cases, when breakpoints of some antimicrobials (pefloxacin, cephalexin, cephradine, colistin, polymyxin B) in CLSI were unavailable, the guideline of European Committee on Antimicrobial Susceptibility Testing (EUCAST) was used[38]. Isolates resistant to at least one agent in three or more antimicrobial classes were defined as multidrug resistant (MDR), while isolates resistant to at least one agent in all but two or fewer antimicrobial classes i.e. bacterial isolates remain susceptible to only one or two classes were defined as extensively drug resistant (XDR)[39]. Moreover, when isolates remained susceptible to three classes were defined as possible extensively drug resistant (pXDR) as per outline given in the report of Magiorakos, Srinivasan (39). The following antimicrobials were tested: penicillins [ampicillin (10 μg)], penicillins + β-lactamase inhibitors [amoxicillin-clavulanic acid (30 μg)], antipseudomonal penicillins + β-lactamase inhibitors [pipercillin-tazobactam (110 μg)], non-extended spectrum cephalosporins [first-generation cephalosporins: cephalexin (30 μg), cephradine (30 μg); second-generation cephalosporins: cefuroxime (30 μg), cefaclor (30 μg)], extended-spectrum cephalosporins [third-generation cephalosporins: cefotaxime (30 μg), ceftriaxone (30 μg), ceftazidime (30 μg), cefixime (5 μg); fourth-generation cephalosporins: cefepime (30 μg)], monobactams [aztreonam (30 μg)], cephamycins [cefoxitin (30 μg)], fluoroquinolones [nalidixic acid (30 μg), ciprofloxacin (5 μg), levofloxacin (5 μg), norfloxacin (10 μg), ofloxacin (5 μg), gatifloxacin (5 μg), pefloxacin (5 μg)], tetracyclines [doxycycline (10 μg)], aminoglycosides [gentamicin (10 μg), amikacin (30 μg)], folate pathway inhibitors [trimethoprim-sulfamethoxazole (25 μg)], glycylcyclines [tigecycline (15 μg)], phenicols [chloramphenicol (30 μg)], polymyxins [colistin, polymyxin B].

**Data analysis**

Descriptive statistics were used to compute the prevalence of bacteria and phenotypic resistance percentage. The significant differences in prevalence of bacteria and resistance percentage among sample types, and study areas were determined by using chi-square test (Z-test for proportions) and Fisher's exact test (if one or more expected cell frequencies were less than 5). The statistical software package SPSS version 22.0 (IBM, Somers, NY) was used for the analyses.

**Spatial mapping**

The spatial mapping was done with district-wise prevalence data of ESBL-*E. coli* and *Salmonella* spp. from 64 live bird markets using QGIS 3.34.8 'Prizren' for desktop (qgis.org).

**Ethics statement**

This study was approved by the Animal Welfare and Experimentation Ethics Committee of Bangladesh Agricultural University, Mymensingh. The approval number was AWEEC/BAU/2017(13). No animal experimentation was done in this study. However, informed written consent was taken from the live bird shop owners before sampling.

**Results**

**Prevalence of ESBL-producing and carbapenem-resistant bacteria**

The prevalence of ESBL-*E. coli* isolated from retail chicken meat and LBM sewage samples was 70% (224/320) and 79.7% (51/64), respectively, while the prevalence of ESBL-*Salmonella* spp. was 43.4% (139/320) in retail chicken meat and 28.1% (18/64) in LBM sewage samples. Furthermore, we found carbapenem-resistant (CR) *E. coli* in 54.1% (173/320) and 46.9% (30/64) of the retail chicken meat and LBM sewage samples, respectively (Fig 2). On the other hand, the prevalence of CR-*Salmonella* spp. was 37.2% (119/320) in retail chicken meat and 12.5% (8/64) in LBM sewage samples.

**Fig 2. Prevalence of ESBL-producing and carbapenem-resistant *E. coli* and *Salmonella* spp. isolated from retail chicken meat and LBM sewage samples.**

**Prevalence of ESBL-encoding and carbapenemase genes**

Among ESBL-encoding genes, three (1.3%) isolates of ESBL-*E. coli* from retail chicken meat and 2 (3.9%) isolates from LBM sewage samples harbored the *bla*CTX-M-1 gene, while 5 (3.6%) isolates of ESBL-*Salmonella* spp. from retail chicken meat were positive for *bla*CTX-M-1 gene (Fig 3 and 4a). The *bla*CTX-M-2 gene was detected in one (2%) ESBL-*E. coli* isolate from LBM sewage samples. The carbapenemase gene, *bla*NDM-1, was detected in 5.2% (9/173) isolates of CR-*E. coli* and 33.6% (40/119) isolates of CR-*Salmonella* spp. from retail chicken meat samples, while none of the CR-*E. coli* and CR-*Salmonella* spp. isolates from LBM sewage samples carried *bla*NDM-1 gene (Fig 3 and Fig 4b).

**Fig 3. Prevalence of resistance genes in ESBL-producing and carbapenem-resistant *E. coli* and *Salmonella* spp. isolated from retail chicken meat and LBM sewage samples.**

**Fig 4a-b. ESBL-encoding and carbapenemase genes in ESBL-producing and carbapenem-resistant *E. coli* and *Salmonella* spp. isolates by uniplex and duplex PCR following 1.5% agarose gel electrophoresis and ethidium bromide staining.** Legends: a) M = DNA marker (100 bp), Lane 1 = Positive control (*bla*CTX-M-1 and *bla*CTX-M-2), Lane 2 = Negative control, Lane 3 = Positive for *bla*CTX-M-2 gene; Lane 4-11 = Positive for *bla*CTX-M-1 gene. b) M = DNA marker (100 bp), Lane 1 = Positive control (*bla*NDM-1), Lane 2 = Negative control, Lane 3-11 = Positive for *bla*NDM-1.

**Spatial distribution of ESBL-producing and CR-*E. coli and Salmonella* spp.**

The district-wise analysis revealed that the prevalence of ESBL-*E. coli* isolated from retail chicken meat was higher in Barishal and Sylhet (90%) districts, followed by Patuakhali and Jashore (85%), and Mymensingh, Bogura and Chattogram (80%) compared with other districts (Fig 5a). Furthermore, the highest prevalence (100%) of ESBL-*E. coli* isolated from LBM sewage samples was observed in Mymensingh, Sherpur, Bogura, Sirajganj, Gazipur, Patuakhali, Sylhet, Khulna and Jashore districts, and lowest in Chattogram (25%), while none was recovered from Rangpur district (Fig 5b).

**Fig 5a-d. Maps showing district-wise distribution of ESBL-*E. coli* and *Salmonella* spp. isolated from retail chicken meat and LBM sewage samples.**

Among the ESBL-*Salmonella* spp. isolated from retail chicken meat, the highest prevalence was observed in Sylhet and Gazipur districts (70%), while the lowest prevalence was recorded in Khulna and Sunamganj districts (20% and 25%, respectively) (Fig 5c). Similarly, analysis of LBM sewage samples revealed the highest prevalence of ESBL-*Salmonella* spp. in Bogura district (70%), whereas the lowest prevalence (25%) was detected in Rangpur, Sirajganj, Tangail, Sunamganj, and Sherpur districts (Fig 5d). Notably, no ESBL-*Salmonella* spp. were detected in LBM sewage samples collected from Chattogram, Cox’s Bazar, Patuakhali, Khulna, and Jashore districts.

District-wise distribution of CR-*E. coli* recovered from retail chicken meat revealed that the prevalence of CR-*E. coli* was highest in Barishal district (80%), followed by Mymensingh and Bogura (75%), and Jashore (70%) districts compared with Cox’s Bazar and Rangpur districts (30%) (Fig 6a). In case of LBM sewage samples, the highest prevalence of CR-*E. coli* was obtained in Mymensingh district (100%), and the lowest in Sirajganj district (25%) (6b). However, CR-*Salmonella* spp. from retail chicken meat, the highest occurrence was observed in Rangpur and Gaibandha districts (65%), followed by Cox’s Bazar (60%) than in Sylhet district (5%) (6c). The isolation rate in LBM sewage samples was highest in Barishal and Gaibandha districts (50%) compared with other districts (6d).

**Fig 6a-d. Maps showing district-wise distribution of CR-*E. coli* and *Salmonella* spp. isolated from retail chicken meat and LBM sewage samples.**

**Multidrug resistance pattern**

A high prevalence of multidrug resistance (MDR) was observed among ESBL-producing and carbapenem-resistant (CR) isolates of *E. coli* and *Salmonella* spp. from both retail chicken meat and live bird market (LBM) sewage samples. Among ESBL-producing isolates from retail chicken meat, MDR was detected in 98.2% (220/224) of *E. coli* and 97.8% (136/139) of *Salmonella* spp. For isolates from LBM sewage, MDR was observed in 92.2% (47/51) of ESBL-*E. coli* and 94.4% (17/18) of ESBL-*Salmonella* spp. (Table 1). Among CR-*E. coli* isolates, MDR prevalence was 98.8% (171/173) in retail chicken meat and 100% (30/30) in LBM sewage samples. Similarly, MDR was detected in 97.5% (116/119) of CR-*Salmonella* spp. from retail chicken meat and 87.5% (7/8) from LBM sewage (Table 2).

**Table 1. Multidrug resistance patterns observed among ESBL-*E. coli* and *Salmonella* spp. isolated from retail chicken meat and LBM sewage samples.**

| **Antimicrobial classes** | **No. (%) of isolates** | | | |
| --- | --- | --- | --- | --- |
|  | **Retail chicken meat** | | **LBM sewage** | |
|  | **ESBL-*E. coli***  **(n = 224)** | **ESBL-*Salmonella* spp.**  **(n = 139)** | **ESBL-*E. coli***  **(n = 51)** | **ESBL-*Salmonella* spp.**  **(n = 18)** |
| 3-5 | 88 (39.3)^a^ | 35 (25.2)^a^ | 21 (41.2)^a^ | 6 (33.3)^a^ |
| 6-8 | 117 (52.2)^a^ | 66 (47.5)^b^ | 22 (43.1)^a^ | 11 (61.1)^b^ |
| ≥ 9 | 15 (6.7)^b^ | 35 (25.2)^a^ | 4 (7.8)^b^ | 0 |
| **MDR** | **220 (98.2)** | **136 (97.8)** | **47 (92.2)** | **17 (94.4)** |
| **pXDR** | **2 (0.9)** | **4 (2.9)** | **0** | **0** |

^ab^Values in the same column with different letters differ significantly (p ≤ 0.05).

**Table 2. Multidrug resistance patterns observed among CR-*E. coli* and *Salmonella* spp. isolated from retail chicken meat and LBM sewage samples.**

| **Antimicrobial classes** | **No. (%) of isolates** | | | |
| --- | --- | --- | --- | --- |
|  | **Retail chicken meat** | | **LBM sewage** | |
|  | **CR-*E. coli***  **(n = 173)** | **CR-*Salmonella* spp.**  **(n = 119)** | **CR-*E. coli***  **(n = 30)** | **CR-*Salmonella* spp.**  **(n = 8)** |
| 3-5 | 65 (37.6)^a^ | 37 (31.1)^a^ | 12 (40.0)^a^ | 2 (25.0)^a^ |
| 6-8 | 92 (53.2)^a^ | 63 (52.9)^b^ | 15 (50.0)^a^ | 5 (62.5)^b^ |
| ≥ 9 | 14 (8.1)^b^ | 16 (13.4)^c^ | 3 (10.0)^b^ | 0 |
| **MDR** | **171 (98.8)** | **116 (97.5)** | **30 (100.0)** | **7 (87.5)** |
| **pXDR** | **2 (1.2)** | **2 (1.7)** | **0** | **0** |

^abc^Values in the same column with different letters differ significantly (p ≤ 0.05).

Notably, in this study, it was observed that, two isolates (0.9%) of ESBL-*E. coli*, 4 isolates (2.9%) of ESBL-*Salmonella* spp., 2 (1.2%) isolates of CR-*E. coli*, and 2 (1.7%) isolates of CR-*Salmonella* spp. from retail chicken meat, were possible extensively drug resistant (pXDR) (Table 1-2).

**Individual antimicrobial resistance pattern**

Isolates of ESBL-*E. coli* (40.6% and 21%) and *Salmonella* spp. (34.5% and 22.3%), as well as CR-*E. coli* (42.8% and 21.4%) and *Salmonella* spp. (42.9% and 25.2%) recovered from retail chicken meat, exhibited resistance to 9–12 and 5–8 antimicrobial agents, respectively (Fig 7). Notably, a considerable number of isolates demonstrated resistance to ≥13 antimicrobial agents, including 65 (29%) of ESBL-*E. coli*, 53 (38.1%) of ESBL-*Salmonella* spp., 53 (30.6%) of CR-*E. coli*, and 34 (28.6%) of CR-*Salmonella* spp.

In isolates obtained from LBM sewage samples, 27.5% of ESBL-*E. coli* and 30% of CR-*E. coli* displayed resistance to 9–12 antimicrobial agents, while 23.5% of ESBL-*E. coli* and 16.7% of CR-*E. coli* were resistant to 5–8 antimicrobials (Fig 7). In contrast, the majority of ESBL-*Salmonella* spp. isolates (38.9%) were resistant to 5–8 agents, whereas CR-*Salmonella* spp. isolates (37.5%) were resistant to 9–12 agents. Importantly, resistance to ≥13 antimicrobial agents was detected in 27.5% of ESBL-*E. coli* and 5.6% of *Salmonella* spp., and in 43.3% of CR-*E. coli* and 12.5% of CR-*Salmonella* spp. isolates.

**Fig 7. Resistance distribution of ESBL-producing and CR -*E. coli* and *Salmonella* spp. to number of antimicrobial agents isolated from retail chicken meat and LBM sewage samples.**

The heat map illustrating the antimicrobial resistance patterns of ESBL-*E. coli* isolated from retail chicken meat revealed the highest resistance to pefloxacin (98.7%), followed by ampicillin (95.5%), trimethoprim-sulfamethoxazole (92.0%), nalidixic acid (79.9%), and doxycycline (75.4%) (Fig 8). High resistance rates were also noted for ciprofloxacin (67.4%), ofloxacin (67.0%), gatifloxacin (66.1%), and levofloxacin (61.6%). The lowest resistance was observed for tigecycline (0.4%) and cefoxitin (2.7%). Resistance to colistin was identified in approximately 39% of isolates. Among ESBL-*Salmonella* spp. from retail chicken meat, the highest resistance was observed for colistin, polymyxin B, and doxycycline (98.6% each), followed by pefloxacin (89.9%), trimethoprim-sulfamethoxazole (89.2%), ampicillin (84.9%), and nalidixic acid (80.6%). In contrast, low resistance was detected for tigecycline (1.4%), piperacillin-tazobactam (4.3%), cefoxitin (9.4%), and aztreonam (11.5%).

In ESBL-*E. coli* isolates recovered from LBM sewage, the highest resistance was noted against pefloxacin (94.1%), trimethoprim-sulfamethoxazole (86.3%), ampicillin (82.4%), amoxicillin-clavulanic acid (62.7%), and doxycycline (60.8%) (Fig 8). The lowest resistance was recorded for cefoxitin (2.0%), piperacillin-tazobactam (3.9%), ceftazidime and polymyxin B (7.8% each), and amikacin (9.8%). Colistin resistance was detected in 37.3% of isolates, while all isolates were susceptible to tigecycline. Regarding ESBL-*Salmonella* spp. isolated from LBM sewage, 94.4% of the isolates exhibited resistance to both colistin and polymyxin B. Additionally, 83.3% were resistant to doxycycline and pefloxacin, and 72.2% to trimethoprim-sulfamethoxazole (Fig 8). Lower resistance was observed for levofloxacin, ceftazidime, and cefoxitin (5.6% each). No resistance was detected against cephalexin, cefaclor, ceftriaxone, cefepime, piperacillin-tazobactam, amikacin, or tigecycline.

**Fig 8. Heat map showing individual antimicrobial resistance pattern of ESBL-*E. coli* and *Salmonella* spp. isolated from retail chicken meat and LBM sewage samples.**

As shown in Fig 9, CR-*E. coli* isolates from both retail chicken meat and LBM sewage exhibited the highest resistance to pefloxacin (100% each), ampicillin (96.0% and 86.7%), trimethoprim-sulfamethoxazole (93.1% and 90.0%), nalidixic acid (84.4% and 76.7%), and doxycycline (75.7% and 73.3%), respectively. For CR-*Salmonella* spp., nearly all isolates were resistant to colistin and polymyxin B (99.2% and 100%), pefloxacin (99.2% and 87.5%), doxycycline (95.8% and 87.5%), nalidixic acid (89.1% and 75.0%), trimethoprim-sulfamethoxazole (88.2% and 62.5%), and ampicillin (80.7% and 62.5%) from retail chicken meat and LBM sewage, respectively.

**Fig 9. Heat map showing individual antimicrobial resistance pattern of CR-*E. coli* and *Salmonella* spp. isolated from retail chicken meat and LBM sewage samples.**

**Discussion**

The present LBM survey documented a comprehensive findings on the magnitude and distribution of ESBL-producing and CR-*E. coli* and *Salmonella* spp. alongside their antimicrobial resistance patterns in retail chicken meat and LBM sewage samples from 64 LBMs of 32 upazilas in 16 districts of all the eight divisions of Bangladesh. Our findings revealed that 70% of *E. coli* and 43.4% of *Salmonella* spp. from retail chicken meat were ESBL producers. These figures are higher than the results of similar studies conducted in India, Istanbul and South Korea, in which the authors reported that 19.1%, 17.8% and 15.8% of retail chicken meat were contaminated with ESBL-producing *E. coli* and *Salmonella* spp., respectively[11, 12, 40], and this may be a potential hazard to the consumers. Similarly, the prevalence of ESBL-producing *E. coli* and *Salmonella* spp. was also very high (79.7%, and 28.1%, respectively) in LBM sewage samples. The higher prevalence in LBM sewage samples suggests that contaminated environments may serve as reservoirs and amplification points for resistant bacteria due to the accumulation of animal waste and poor hygiene practices[41]

Concerning carbapenem resistance, our investigation identified 54.1% CR-*E. coli* in retail chicken meat, and 46.9% in LBM sewage samples. These findings are alarming because carbapenems are last-resort antibiotics in human medicine, and the presence of carbapenem resistant strains in the food chain represents a serious risk to public health[16]. The prevalence of CR-*Salmonella* spp., although at slightly lower rates­­­­ — 37.2% in retail chicken meat and 12.5% in LBM sewage—is also alarming. Carbapenem resistance in *Salmonella* spp. is rare but growing, and it is frequently linked to plasmid-mediated carbapenemase genes, including *bla*KPC, *bla*NDM-1, and *bla*OXA-48[16].

The higher prevalence of ESBL-producing and CR-*E. coli* and *Salmonella* spp. isolated from retail chicken meat and LBM sewage samples could be attributed to contamination during processing, transport, and handling, which promotes cross-contamination and bacterial multiplication[42]. Furthermore, the extensive use of antibiotics in livestock and poultry production, especially in countries with limited regulatory oversight, contributes considerably to the emergence and dissemination of AMR pathogens[14].

The findings of this study highlight the presence of ESBL-encoding and carbapenemase genes in *E. coli* and *Salmonella* spp. isolated from retail chicken meat and LBM sewage samples, underscoring significant public health concerns regarding the dissemination of AMR determinants through the food chain and environmental reservoirs. Among the ESBL-producing isolates, the *bla*CTX-M-1 gene was detected in 1.3% and 3.6% of *E. coli* and *Salmonella* spp. from retail chicken meat, as well as in 3.9% isolates of *E. coli* from LBM sewage, in contrast to prior reports in Turkey and South Korea, where this gene was detected in 41.9% of *E. coli* and 100% of *Salmonella* spp., respectively[43, 44]. The CTX-M-type β-lactamases, especially those of the CTX-M-1 group, are the most prevalent ESBL enzymes globally in both clinical and non-clinical contexts, including food animals and their associated environments[10]. The presence of *bla*CTX-M-1 in retail chicken meat is particularly concerning, given the potential for transmission to humans through food handling and consumption[45]. Interestingly, the *bla*CTX-M-2 gene was only detected in a single (2%) *E. coli* isolate from LBM sewage, indicating a more limited distribution of this gene variant in the studied environment.

The presence of the carbapenemase gene *bla*NDM-1 in 5.2% of CR-*E. coli* and a substantially higher proportion (33.6%) of CR-*Salmonella* spp. from retail chicken meat indicates the alarming development of carbapenem resistance in foodborne pathogens. The *bla*NDM-1 gene produces a potent enzyme that is capable of hydrolyzing almost all β-lactams antibiotics, including carbapenems, which are often considered as an antibiotics of last resort[16]. The finding of *bla*NDM-1 gene in retail chicken meat samples, and its absence in LBM sewage, may indicate distinct selective pressures and contamination pathways in meat processing environments compared to wastewater systems.

Distribution of ESBL-producing and CR-*E. coli* and *Salmonella* spp. in retail chicken meat and LBM sewage samples were found to vary from district to district. For retail chicken meat, the present study revealed the higher prevalence of ESBL-*E. coli* in Barishal and Sylhet districts than in other districts. The distribution of ESBL-*Salmonella* spp. in retail chicken meat has also been observed to be varied between districts, with the highest prevalence in Sylhet and Gazipur districts, and the least in Khulna and Sunamganj districts. Earlier studies reported that a variable prevalence of ESBL-*E. coli* and *Salmonella* spp. were detected from frozen chicken meat and cloacal swab of domestic chickens in Dhaka, Chattogram, Sylhet, and Rajshahi district of Bangladesh[15, 21-23]. This variation may reflect regional disparities in poultry farming practices and antimicrobial usage in Bangladesh[4]. On the other hand, ESBL-*E. coli* and *Salmonella* spp. in LBM sewage samples were more frequently distributed in almost all the districts except Rangpur, Chattogram, Cox’s Bazar, Patuakhali, Jashore and Khulna districts, where ESBL-*E. coli* and *Salmonella* spp. was not recovered. Previous studies have been reported high levels of ESBL-*E. coli* in household water supply and sewage water from different areas of Dhaka district, suggesting localized variations in environmental contamination and waste management[23, 24]. The present study also observed that the prevalence of CR-*E. coli* and *Salmonella* spp. in retail chicken meat and LBM sewage samples varied between the districts, which might be due to differences in the degree of chicken processing in retail stores and the hygiene practices of slaughterers or workers at different LBMs stalls. Since different LBMs are anticipated to use different management techniques while slaughtering of chickens, and processing of chicken meat, there are varying hazards associated with the presence of CR-*E. coli* and *Salmonella* spp. in retail chicken meat. The processes involved in slaughtering, plucking, defeathering, and freezing the chicken meat for retail sale could also result in contamination[18, 46].

An important finding of concern is that more than 90% of ESBL-*E. coli* and *Salmonella* spp. from retail chicken meat and LBM sewage samples, and around 98% CR-*E. coli* and *Salmonella* spp. from retail chicken meat, and 100% CR-*E. coli* and 87.5% CR-*Salmonella* spp. from LBM sewage samples were MDR. A significantly higher percentage of MDR was observed in the present study consistent with other previous study, in which 100% of ESBL-*E. coli* recovered from retail chicken meat in Tanzania[19], were reported as MDR. This scenario is especially alarming in low and middle-income countries like Bangladesh since antibiotic resources are limited, and treatment options are compromised when strains are resistant to several classes of antibiotics. Of note, the current study also observed that 0.9% of ESBL-*E. coli*, 2.9 % of ESBL-*Salmonella* spp., 1.2% of CR-*E. coli* and 1.7% of CR-*Salmonella* spp. in retail chicken meat were pXDR. A study from Pakistan reported that the presence of an extensively drug resistant *Salmonella enterica* in retail chicken meat[47]. The occurrence of high MDR and existance of pXDR among ESBL-producing and CR-*E. coli* and *Salmonella* spp. could be due to widespreed and unrestricted use of antibiotics during poultry production in Bangladesh as well as low levels of waste management systems, which increases selective pressure for MDR bacteria, thus facilitating the emergence and dissemination of MDR foodborne bacteria in poultry production system and retail poultry products. To reduce MDR foodborne bacterial contamination in retail chicken meat, maintaining adequate hygiene during and after chicken slaughter is very essential.

The emergence of AMR among foodborne bacterial isolates, particularly ESBL-producing and CR-*E. coli* and *Salmonella* spp., pose a significant public health issue that requires ongoing surveillance. Several studies have reported a substantial rise in AMR among foodborne bacteria, which has led to an increase in foodborne diseases[2, 48]. Resistant bacteria can exist in a silent carrier state and later lead to foodborne infections that may go unrecognized as being foodborne in origin[2]. The present study observed that a noticeable number of isolates of ESBL-*E. coli* (47.3%, and 33.3%), and ESBL-*Salmonella* spp. (46%, and 16.7%), as well as CR-*E. coli* (30.6% and 43.3%) and CR-*Salmonella* spp. (28.6% and 12.5%) in retail chicken meat and LBM sewage, respectively, demonstrated resistance to more than 13 antimicrobial agents. These findings align with previous reports that have documented high levels of AMR in bacterial isolates from retail chicken meat [11, 12, 19, 40, 48]. This widespread resistance can be attributed, in part, to the extensive use of antibiotics in poultry farming for therapeutic purposes, growth promotion, and disease prevention.[2]. Furthermore, antimicrobial-resistant foodborne bacteria could be transmitted to other individuals, the food production chain, and the environment.

The highest resistance was observed to fluoroquinolones, mainly pefloxacin and nalidixic acid in ESBL-prodicing as well as CR-*E. coli* and *Salmonella* spp. from both types of samples, which is consistent with other studies in retail chicken meat[2, 11, 12, 40, 48, 49]. The high resistance rates observed in this study should be considered because these antibiotics are critically important antibiotics for human health[50]. According to the results, ampicillin, trimethoprim-sulfamethoxazole, and doxycycline resistance was also observed very high in ESBL-producing and CR-*E. coli* and *Salmonella* spp. isolated from both types of samples, which unfortunately are the most commonly used antibiotics in both humans and animals. Similar high resistance rates were also reported for these antibiotics in several other studies conducted in India[11], South Korea[12]; Tanzania[2], Pakistan[48] Istanbul[40], Senegal[49]. The marked resistance to these antimicrobials is perhaps not surprising because these drugs are inexpensive, and are available from diverse sources where they are sold with or without prescription and the most commonly used in poultry farms. It has been demonstrated that limiting the use of antibiotics reduces AMR in poultry and is likely to lower AMR in humans, even if the magnitude of the effect has not yet been determined[2]. Additionally, Bangladesh should control the use of antibiotics in poultry in order to reduce the levels of resistance found in the current study.

In the present study, a higher percentage isolates of ESBL-*E. coli* (39.3%, and 37.3%), ESBL-*Salmonella* spp. (98.6%, and 94.4%), and CR-*Salmonella* spp. in retail chicken meat and LBM sewage samples, respectively showed resistance to colistin, last-resort antimicrobials used to treat human infections caused by carbapenem-resistant bacteria. This high resistance rates of ESBL-*E. coli* and *Salmonella* spp. in retail chicken meat as well as in LBM sewage samples reflect its widespread use in poultry feed in Bangladesh. Recently, Bangladesh government banned the manufacture, sale and distribution of colistin and its formulations for poultry production to tackle colistin resistance but pack size of 1 L and above is still permitted to use in breeder flocks in Bangladesh.

On the other hand, a relatively low resistance rate to tigecycline, and cefoxitin was observed in ESBL-*E. coli* and *Salmonella* spp. in both retail chicken meat and LBM sewage samples. Our result is in agreement with findings of other studies[12, 40]. These results might be due to the fact that in Bangladesh, tigecycline and cefoxitin antibiotics are not common in veterinary medicine, especially in food-producing animals such as poultry.

It would be worthwhile if samples were taken from slaughterer or workers. However, retail chicken meat samples were collected from 64 LBMs of 32 upazilas of 16 districts in all the divisions of Bangladesh; thus, the data represent the scenario of whole Bangladesh. Further work should be performed to characterize ESBL-producing and CR-*E. coli* and *Salmonella* spp. isolates of poultry and human origin from the same region sharing the same resistance markers in order to highlight potential horizontal gene transfer by these foodborne organisms.

**Conclusion**

The present study documented, for the first time, a comprehensive finding on the prevalence and distribution of ESBL-producing and CR-*E. coli* and *Salmonella* spp. along with their antimicrobial resistance pattern in retail chicken meat and LBM sewage samples covering 16 districts of eight divisions of Bangladesh. The results highlight the role of contaminated retail chicken meat as potential sources of ESBL-producing and CR-*E. coli* and *Salmonella* spp.; strains that may spread to humans and into the environment. These results raise serious concerns regarding public health and food safety because retail chicken meat could serve as a reservoir for multidrug resistant bacteria, which could be transferred to humans through the food chain. This study also highlighting the significance of implementing sanitary handling measures to limit microbial contamination in retail meat as well as consumer education is required to ensure safe handling and preparation of meat products. Thus, the findings of the present study should be used to emphasize the responsibility of veterinarians and relevant authorities to develop prudent antimicrobial use guidelines to minimize the emergence and spread of AMR in bacterial strains originating from retail chicken meat. The drug regularity authority of Bangladesh should develop an implementable monitoring and evaluation system to tackle this issue. Besides, alternative therapies should be explored and assessed for treating and preventing multidrug resistant bacterial infections. Our research may serve as a baseline for future interventions aimed at limiting the spread of AMR in the environment.

**Acknowledgments**

The authors would like to thank the Bangabandhu Science and Technology Fellowship Trust for giving the fellowship. The authors would also like to thank the Department of Medicine, Faculty of Veterinary Science, Bangladesh Agricultural University, Mymensingh for the support during the research.

**Author contributions**

Conceptualization: Md. Taohidul Islam; Methodology: Mst. Sonia Parvin, Sudipta Talukder, Sayra Tasnin Sharmy, Md. Mehedi Hasan; Formal analysis and investigation: Mst. Sonia Parvin, Md. Taohidul Islam; Writing - original draft preparation: Mst. Sonia Parvin; Writing - review and editing: Sudipta Talukder, Sayra Tasnin Sharmy, Md. Mehedi Hasan, Md. Taohidul Islam; Funding acquisition: Md. Taohidul Islam; Resources: Sudipta Talukder, Sayra Tasnin Sharmy, Md. Mehedi Hasan; Supervision: Md. Taohidul Islam. All authors read and approved the final manuscript.

Data availability

The datasets generated and analyzed during the current study are available from the corresponding author on reasonable request.

**Funding**

This work was partially supported by the BAS-USDA Program in Agriculture and Life Sciences [grant number BAS-USDA PALS LS-18] and Ministry of Science and Technology of Bangladesh [grant number SRG-221102]. The funder had no role in the design of the study; in the collection, analysis, or interpretation of data; in the writing of the manuscript; or in the decision to publish the results.

**Competing interests**

The authors declare no competing interests.

**References**

1.	O’Neil J. Review on antibiotic resisitance. Antimicrobial resistance: tackling a crisis for the health and wealth of nations London. 2014:1-16.

2.	Kim J, Ahn J. Emergence and spread of antibiotic-resistant foodborne pathogens from farm to table. Food Sci Biotechnol. 2022;31(12):1481-99. Epub 20220901. doi: 10.1007/s10068-022-01157-1. PubMed PMID: 36065433; PubMed Central PMCID: PMCPMC9435411.

3.	Van Boeckel TP, Brower C, Gilbert M, Grenfell BT, Levin SA, Robinson TP, et al. Global trends in antimicrobial use in food animals. Proc Natl Acad Sci. 2015;112(18):5649-54. Epub 03/19. doi: 10.1073/pnas.1503141112. PubMed PMID: 25792457.

4.	Hasan B, Faruque R, Drobni M, Waldenström J, Sadique A, Ahmed KU, et al. High prevalence of antibiotic resistance in pathogenic *Escherichia coli* from large- and small-scale poultry farms in Bangladesh. Avian Dis. 2011;55(4):689-92.

5.	Banik S, Sultana R, Hoque MN. Prevalence and molecular characterization of multidrug-resistant *Salmonella* spp. in retail poultry meat in live bird markets of Dhaka, Bangladesh. Microbial Drug Resistance. 2022;28(7):867–76.

6.	World Health Organization. WHO guidelines on use of medically important antimicrobials in food-producing animals 2017. Available from: <https://www.who.int/publications/i/item/9789241550130>.

7.	Blaak H, Lynch G, Italiaander R, Hamidjaja RA, Schets FM, de Roda Husman AM. Multidrug-resistant and extended spectrum beta-lactamase-producing Escherichia coli in Dutch surface water and wastewater. PLoS One. 2015;10(6):e0127752.

8.	Nhung NT, Chansiripornchai N, Carrique-Mas JJ. Antimicrobial resistance in bacterial poultry pathogens: A review. Front Vet Sci. 2017;4:126-. doi: 10.3389/fvets.2017.00126. PubMed PMID: 28848739.

9.	Logan LK, Weinstein RA. The epidemiology of carbapenem-resistant Enterobacteriaceae: the impact and evolution of a global menace. The Journal of infectious diseases. 2017;215(suppl_1):S28-S36.

10.	Bush K, Bradford PA. Epidemiology of β-Lactamase-Producing Pathogens. Clin Microbiol Rev. 2020;33(2). Epub 20200226. doi: 10.1128/cmr.00047-19. PubMed PMID: 32102899; PubMed Central PMCID: PMCPMC7048014.

11.	Batabyal K, Banerjee A, Dey S, Samanta I, Isore DP, Singh AD. Detection and characterization of multi drug-resistant extended-spectrum and pAmpC beta-lactamases producing *Escherichia coli* from chicken meat in West Bengal, India. Int J Curr Microbiol Appl Sci. 2020;9(7):80-9.

12.	Jeon HY, Seo KW, Kim YB, Kim DK, Kim SW, Lee YJ. Characteristics of third-generation cephalosporin-resistant *Salmonella* from retail chicken meat produced by integrated broiler operations. Poult Sci. 2019;98(4):1766-74. doi: <https://doi.org/10.3382/ps/pey514>.

13.	Boucher HW, Talbot GH, Bradley JS, Edwards JE, Gilbert D, Rice LB, et al. Bad bugs, no drugs: no ESKAPE! An update from the Infectious Diseases Society of America. Clinic Infect Dis. 2009;48(1):1-12.

14.	Van Boeckel TP, Pires J, Silvester R, Zhao C, Song J, Criscuolo NG, et al. Global trends in antimicrobial resistance in animals in low-and middle-income countries. Proc Natl Acad Sci. 2015;112(18):5649–54.

15.	Parvin MS, Hasan MM, Ali MY, Chowdhury EH, Rahman MT, Islam MT. Prevalence and multidrug resistance pattern of *Salmonella* carrying extended-spectrum β-lactamase in frozen chicken meat in Bangladesh. J Food Prot. 2020;83:2107–21. doi: 10.4315/jfp-20-172.

16.	van Duin D, Doi Y. The global epidemiology of carbapenemase-producing *Enterobacteriaceae*. Virulence. 2017;8(4):460-9. Epub 20160811. doi: 10.1080/21505594.2016.1222343. PubMed PMID: 27593176; PubMed Central PMCID: PMCPMC5477705.

17.	Sayeed MA, Smallwood C, Imam T, Mahmud R, Hasan RB, Hasan M, et al. Assessment of hygienic conditions of live bird markets on avian influenza in Chittagong metro, Bangladesh. Prev Vet Med. 2017;142:7-15.

18.	Olsen RH, Bisgaard M, Lohren U, Robineau B, Christensen H. Extended spectrum β-lactamase-producing *Escherichia coli* isolated from poultry: a review of current problems, illustrated with some laboratory findings. Avian Pathol. 2014;43:199-208.

19.	Omar M, Kilale AM, Ally MS, Rashid HK, Gimbi AA. Prevalence of multidrug resistant extended spectrum β-lactamase producing *E. coli* in live chicken and chicken products in Zanzibar. PAMJ - One Health. 2024;13(1). doi: 10.11604/pamj-oh.2024.13.1.40271.

20.	Nordmann P, Naas T, Poirel L. Global spread of carbapenemase-producing Enterobacteriaceae. Emerg Infect Dis. 2011;17(10):1791.

21.	Parvin MS, Talukder S, Ali MY, Chowdhury EH, Rahman MT, Islam MT. Antimicrobial resistance pattern of *Escherichia coli* isolated from frozen chicken meat in Bangladesh. Pathogens. 2020;9(6):420.

22.	Hasan B, Sandegren L, Melhus A, Drobni M, Hernandez, Waldenstrom J, et al. Antimicrobial drug resistant *Escherichia coli* in wild birds and free-range poultry, Bangladesh. Emerg Infect Dis. 2012;18(12):2055-8.

23.	Parvin MS, Ali MY, Mandal AK, Talukder S, Islam MT. Sink survey to investigate multidrug resistance pattern of common foodborne bacteria from wholesale chicken markets in Dhaka city of Bangladesh. Sci Rep. 2022;12(1):10818.

24.	Talukdar PK, Rahman M, Rahman M, Nabi A, Islam Z, Hoque MM, et al. Antimicrobial resistance, virulence factors and genetic diversity of *Escherichia coli* isolates from household water supply in Dhaka, Bangladesh. PLoS One. 2013;8(4):e61090.

25.	Mahmud ZH, Kabir MH, Ali S, Moniruzzaman M, Imran KM, Nafiz TN, et al. Extended-spectrum beta-lactamase-producing *Escherichia coli* in drinking water samples from a forcibly displaced, densely populated community setting in Bangladesh. Front Public Health. 2020;8:228.

26.	Parvez M, Khasru A, Mou TJ, Ahmed F. Extended spectrum beta-lactamase (ESBL) producing *Enterobacteria* in aquatic environmental sources of Bangladesh. Int Biol Biomed J. 2017;3(1):21-4.

27.	Thrusfield M, Christley R. Veterinary Epidemiology. 4th ed: Wiley-Blackwell; 2018.

28.	Islam MK, Kabir SL, Haque AZ, Sarker Y, Sikder M. Molecular detection and characterization of *Escherichia coli*, *Salmonella* spp. and *Campylobacter* spp. isolated from broiler meat in Jamalpur, Tangail, Netrokona and Kishoreganj districts of Bangladesh. Afr J Microbiol Res. 2018;12(32):761-70.

29.	Rahman MA, Rahman A, Islam MA, Alam MM. Detection of multidrug resistant *Salmonella* from milk and meat in Bangladesh. Bangl J Vet Med. 2018;16(1):115-20.

30.	ISO. ISO standards catalogue 07.100.30. Food Microbiol. <http://www.iso.org/iso/products/standards/catalogue_ics_browse.htm?ICS1=07&ICS2=100&ICS3=30> 2011.

31.	Dashti A, Jadaon M, Abdulsamad A, Dashti H. Heat treatment of bacteria: a simple method of DNA extraction for molecular techniques. Kuwait Med J. 2009;41(2):117-22.

32.	Wang RF, Cao WW, Cerniglia CE. PCR detection and quantitation of predominant anaerobic bacteria in human and animal fecal samples. Appl Environ Microbiol. 1996;62(4):1242-7.

33.	Chiu TH, Chen TR, Hwang WZ, Tsen HY. Sequencing of an internal transcribed spacer region of 16S-23S rRNA gene and designing of PCR primers for the detection of *Salmonella* spp. in food. Int J Food Microbiol. 2005;97(3):259-65. doi: <https://doi.org/10.1016/j.ijfoodmicro.2004.04.005>.

34.	CLSI. Performance standards for antimicrobial susceptibility testing. Wayne, USA: 2018.

35.	Le QP, Ueda S, Nguyen TNH, Dao TVK, Van Hoang TA, Tran TTN, et al. Characteristics of extended-spectrum β-lactamase producing *Escherichia coli* in retail meats and shrimp at a local market in Vietnam. Foodborne Pathog Dis. 2015;12(8):719-25.

36.	Centers for Disease Control and Prevention. Antibiotic/AMR. Available from: <https://www.cdc.gov/>

nhsn/pdfs/datastat/2020-au-report-508.pdf. 2020.

37.	Islam MA, Talukdar PK, Hoque A, Huq M, Nabi A, Ahmed D, et al. Emergence of multidrug-resistant NDM-1-producing Gram-negative bacteria in Bangladesh. European Journal of Clinical Microbiology & Infectious Diseases. 2012;31(10):2593-600. doi: 10.1007/s10096-012-1601-2.

38.	EUCAST. Breakpoint tables for interpretation of MICs and zone diameters. 2018.

39.	Magiorakos A-P, Srinivasan A, Carey RB, Carmeli Y, Falagas ME, Giske CG, et al. Multidrug-resistant, extensively drug-resistant and pandrug-resistant bacteria: an international expert proposal for interim standard definitions for acquired resistance. Clin Microbiol Infect. 2012;18(3):268-81.

40.	Aydin A, Suleymanoglu AA, Abdramanov A, Paulsen P, Dumen E. Detection of extended spectrum ß-lactamase-producing *Escherichia coli* with biofilm formation from chicken meat in Istanbul. Foods. 2024;13(7):1122.

41.	Zurfluh K, Nüesch-Inderbinen M, Morach M, Zihler Berner A, Hächler H, Stephan R. Extended-spectrum-β-lactamase-producing *Enterobacteriaceae* isolated from vegetables imported from the Dominican Republic, India, Thailand, and Vietnam. Appl Environ Microbiol. 2015;81(9):3115-20. Epub 20150227. doi: 10.1128/aem.00258-15. PubMed PMID: 25724954; PubMed Central PMCID: PMCPMC4393435.

42.	Abdel-Kader F, Hamza E, Abdel-Moein KA, Sabry MA. Retail chicken giblets contaminated with extended-spectrum cephalosporin- and carbapenem-resistant *Salmonella enterica* carrying *bla*CMY-2. Vet World. 2022;15(5):1297-304. Epub 20220525. doi: 10.14202/vetworld.2022.1297-1304. PubMed PMID: 35765473; PubMed Central PMCID: PMCPMC9210848.

43.	Baran A, Adigüzel MC, Yüksel M. Prevalence of antibiotic-resistant and extended-spectrum beta-lactamase-producing *Escherichia coli* in chicken meat from Eastern Turkey. Pak Vet J Published online: May 19, 2020 1029261/pakvetj/2020047. 2020.

44.	Chon J-W, Jung H-I, Kuk M, Kim Y-J, Seo K-H, Kim S-K. High occurrence of extended-spectrum β-lactamase-producing *Salmonella* in broiler carcasses from poultry slaughterhouses in South Korea. Foodborne Pathog Dis. 2015;12(3):190-6.

45.	Lazarus B, Paterson DL, Mollinger JL, Rogers BA. Do human extraintestinal *Escherichia coli* infections resistant to expanded-spectrum cephalosporins originate from food-producing animals? A systematic review. Clinic Infect Dis. 2015;60(3):439-52.

46.	Sabry MA, Abdel-Moein KA, Abdel-Kader F, Hamza E. Extended-spectrum β-lactamase-producing *Salmonella* serovars among healthy and diseased chickens and their public health implication. J Glob Antimicrob Resist. 2020;22:742-8.

47.	Asif M, Rahman H, Qasim M, Khan TA, Ullah W, Jie Y. Molecular detection and antimicrobial resistance profile of zoonotic *Salmonella enteritidis* isolated from broiler chickens in Kohat, Pakistan. J Chin Med Assoc. 2017;80(5):303-6. doi: 10.1016/j.jcma.2016.11.007.

48.	Zainab L, Ibrar K, Sadiq A, Hamid AK, Ullah M, Noor R. Extended spectrum beta lactamases-producing *Escherichia coli* in retail chicken meat from Khyber Pakhtunkhwa, Pakistan. Saudi J Biol Sci. 2022;29(6):103280. doi: <https://doi.org/10.1016/j.sjbs.2022.103280>.

49.	Assoumy MA, Bedekelabou AP, Teko-Agbo A, Ossebi W, Akoda K, Nimbona F, et al. Antibiotic resistance of *Escherichia coli* and *Salmonella* spp. strains isolated from healthy poultry farms in the districts of Abidjan and Agnibilékrou (Côte d’Ivoire). Vet World. 2021;14(4):1020.

50.	WHO. Critically important antimicrobials for human medicine. 2019 924151552X.
